# The Cyclin Cln1 Controls Polyploid Titan Cell Formation following a Stress-Induced G_2_ Arrest in Cryptococcus

**DOI:** 10.1128/mBio.02509-21

**Published:** 2021-10-12

**Authors:** Sophie Altamirano, Zhongming Li, Man Shun Fu, Minna Ding, Sophie R. Fulton, J. Marina Yoder, Vy Tran, Kirsten Nielsen

**Affiliations:** a Department of Microbiology and Immunology, University of Minnesota, Minneapolis, Minnesota, USA; Washington University School of Medicine

**Keywords:** *Cryptococcus neoformans*, aneuploidy, cell cycle, cryptococcal meningitis, cryptococcosis, cyclins, *deneoformans*, ploidy, polyploid, polyploidy, titan cell

## Abstract

The pathogenic yeast Cryptococcus neoformans produces polyploid titan cells in response to the host lung environment that are critical for host adaptation and subsequent disease. We analyzed the *in vivo* and *in vitro* cell cycles to identify key aspects of the C. neoformans cell cycle that are important for the formation of titan cells. We identified unbudded 2C cells, referred to as a G_2_ arrest, produced both *in vivo* and *in vitro* in response to various stresses. Deletion of the nonessential cyclin Cln1 resulted in overproduction of titan cells *in vivo* and transient morphology defects upon release from stationary phase *in vitro*. Using a copper-repressible promoter *P_CTR4_-CLN1* strain and a two-step *in vitro* titan cell formation assay, our *in vitro* studies revealed Cln1 functions after the G_2_ arrest. These studies highlight unique cell cycle alterations in C. neoformans that ultimately promote genomic diversity and virulence in this important fungal pathogen.

## INTRODUCTION

Cell division during mitosis typically involves a single round of DNA replication and then equal partitioning of this DNA into two separate, genetically identical cells. Despite the highly regulated process of cell division, cell cycle alterations that produce whole-genome duplications occur naturally throughout the tree of life (1). These whole-genome duplications produce polyploids, which are cells with increased DNA content beyond the typical ploidy state of the organism. Polyploidy is a normal part of development, tissue homeostasis, and a stress response in many multicellular eukaryotes (2). For example, trophoblast giant cells found in mammalian placenta duplicate their genomes while bypassing mitosis and cytokinesis, a process known as endocycling, to form polyploid cells that can exceed 512C (3). Studies in *Drosophila* show that puncture wound healing of the epithelium involves cell fusion and endocycling, both of which produce polyploid cells (4). Additionally, keratinocytes are known to polyploidize, possibly increasing the potential for cellular survival during UV exposure while decreasing the oncogenic potential of the cells (5–8). Unfortunately, while evidence for polyploidy across many kingdoms of life has been identified, the biological function of polyploidy in many cases is still unknown.

Polyploid cells are often not stable and can undergo chromosomal loss or aberrant mitosis to produce cells with an abnormal number of chromosomes that are not an integer multiple of the base genome, known as aneuploid cells (9, 10). The ability to form polyploid and aneuploid cells can be beneficial to an organism but can also make an organism more susceptible to disease. Polyploidy is often associated with the formation and progression of cancer cells due to this ability to generate aneuploid cells. Approximately 90% of solid tumors and 75% of hematopoietic cancers are aneuploid (11), with recent models suggesting that these aneuploid cells arise from polyploid precursors (9, 12–14).

The molecular mechanisms underlying polyploid division and how polyploidy contributes to genomic instability still remain largely unknown. A better understanding of this process would provide insight into the pathological role of polyploidy. The study of polyploidy has generally been constrained to multicellular organisms and genetically engineered yeast. Polyploidy in multicellular organisms is a complex multifactorial process involving temporal and tissue-specific cues that make replicating these processes in a laboratory setting challenging (8). Previous studies in yeast have been very helpful to define the genomic instability of polyploids but required genetically modified strains to induce and study polyploidy (15–17). Recently, the human-pathogenic yeast Cryptococcus neoformans was found to produce enlarged, polyploid cells, known as titan cells, during pulmonary infections (18, 19).

C. neoformans is a haploid (1C) budding yeast with cells that are typically 5 to 7 μm in cell diameter. In contrast, the titan cells have ploidies ranging from 4C to 312C and are 10 to 100 μm in cell diameter (18, 19). Titan cells undergo cell division to produce genetically distinct 1C or aneuploid daughter cell populations that are typical-sized and exhibit increased stress resistance, suggesting that the production of titan cells during infection promotes rapid adaptation to the host environment (20). In addition, titan cells have cell wall changes that impact the host immune response (21, 22). Due to the vast size difference between typical and titan cells, these C. neoformans cells can be readily separated and are a good model system to study the mechanism of naturally occurring polyploid cell formation and subsequent error-prone cell division that leads to genetic variation.

Here, we characterize the C. neoformans cell cycle during infection and *in vitro* to identify how regulation of the cell cycle in C. neoformans leads to titan cell formation and the resulting polyploidy. Using homology to known cyclins and cyclin-dependent kinases (CDKs), we identified the cyclin, Cln1, as a master regulator of titan cell formation. We show C. neoformans has a G_2_ arrest with no bud formation in response to the murine pulmonary environment and also under *in vitro* nutrient deprivation conditions. We show that Cln1 is critical for balancing DNA replication and cell division after the G_2_ arrest. When *CLN1* expression is low, polyploid titan cells are produced in response to specific environmental stimuli. Taken together, these studies identify Cln1 as a master regulator underlying ploidy changes in C. neoformans and highlight the versatility of this unicellular eukaryotic microorganism to understand the molecular regulation and biological function of ploidy changes.

## RESULTS

### C. neoformans has an unbudded G_2_ arrest during *in vivo* infection and *in vitro* nutrient deprivation.

Cell cycle progression is often correlated with cell size and shape (23); thus, cell cycle changes likely precede the observable size and morphology changes that produce the titan cells. The mitotic cell cycle is typically divided into four phases: growth 1 (G_1_), prior to DNA replication; synthesis (S), when the DNA is replicated; growth 2 (G_2_), prior to cell division; and mitosis (M), when cytokinesis occurs and the cell divides. C. neoformans is a budding yeast, similar to the model yeast Saccharomyces cerevisiae. Previous studies in S. cerevisiae linked distinct cell morphologies with the four cell cycle phases (23). In S. cerevisiae, G_1_ cells are unbudded and have a 1C DNA content. A bud appears at the beginning of S phase and grows as the DNA is replicated. Entry into G_2_ occurs when DNA synthesis is completed, and the daughter cell is approximately half the size of the mother cell. M phase involves cytoskeletal tubulin rearrangements to generate the mitotic spindle that pulls the replicated DNA into the daughter cell (24). Similar cell morphology and ploidy changes associated with cell cycle phases were previously shown to occur in log-phase cultures of C. neoformans *in vitro* (25, 26) and are consistent with a cell cycle progression similar to that in S. cerevisiae.

**FIG 1:**
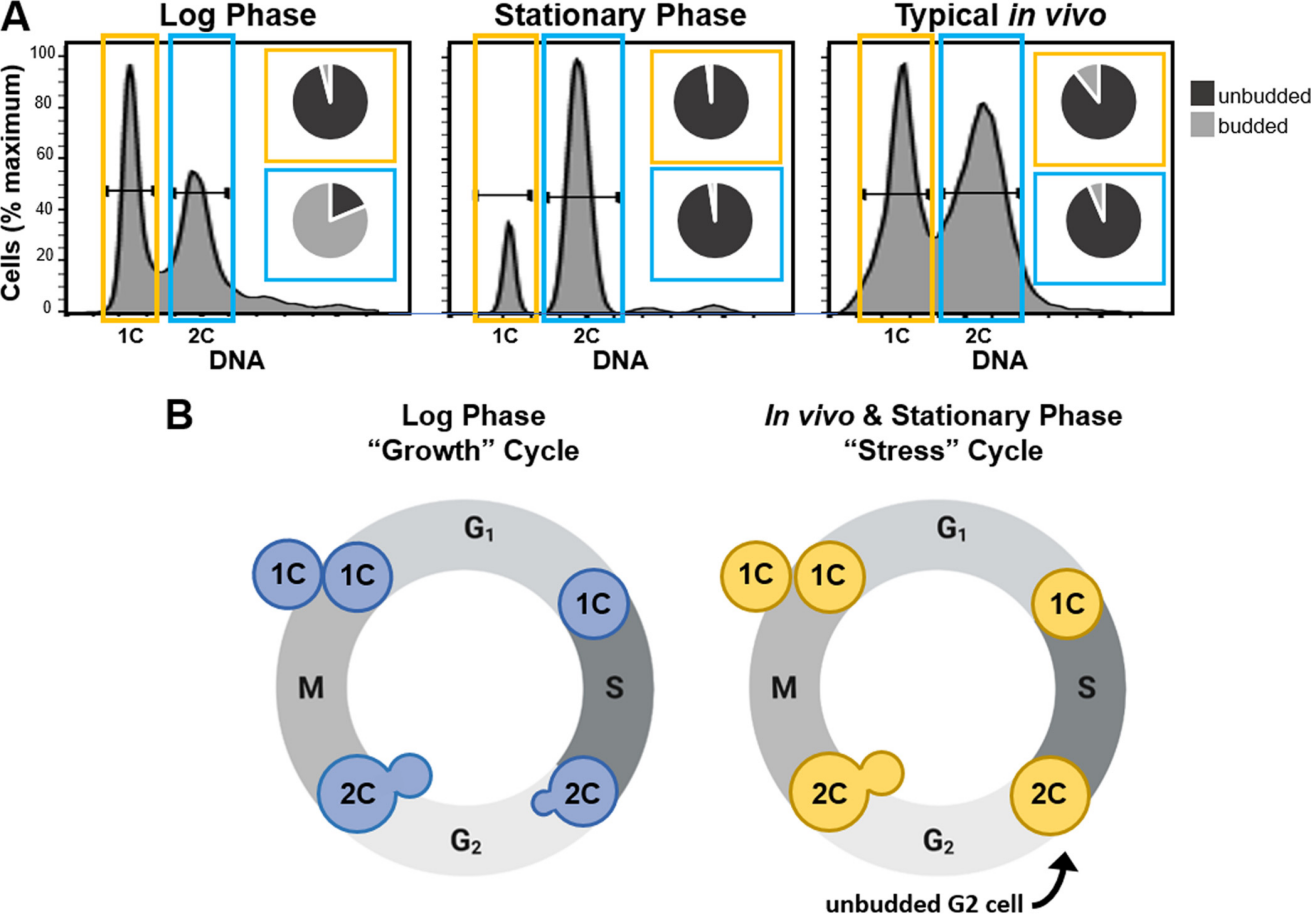
*In vivo* typical sized cells and *in vitro* stationary-phase cells are primarily unbudded 2C cells. (A) Analysis of cell morphology with 1C (yellow) and 2C (blue) DNA content in log-phase cells, stationary-phase cells, and typical-size cells isolated from the lungs of mice at 14 days postinfection (referred to as typical *in vivo*). Cells were grown in the indicated conditions, fixed, and stained with propidium iodide, and then fluorescence-activated cell sorting (FACS) was performed to purify the 1C and 2C cell populations. The resulting purified cells were then analyzed microscopically to determine the proportion of cells containing buds (insets). (B) Schematic representation of the C. neoformans growth cell cycle (blue cells) observed in log-phase cells and the putative stress cell cycle (yellow cells) that is notable for production of unbudded 2C cells that were observed in both stationary-phase cultures *in vitro* and among typical sized cells *in vivo*.

Our previous study showed that *in vivo* typical-sized cells were primarily 2C (18), instead of the predicted mixed population of 1C and 2C cells observed in log-phase cultures grown *in vitro*. To define the *in vivo* cell cycle that precedes titan cell formation, we isolated typical-sized cells based on DNA content by fluorescence-activated cell sorting (FACS), assessed the morphology of each sorted population, and analyzed the ploidy and morphology compared to that of log-phase and stationary-phase cells grown *in vitro* (Fig. 1A). As shown previously (26), our C. neoformans log-phase cells consisted of 1C and 2C peaks, where the majority of the 1C population was unbudded (∼96%, *n* = 226) and the majority of the 2C population was budded (∼81%, *n* = 230) (Fig. 1A). Also consistent with previous studies (26, 27), the stationary-phase cells generated via nutrient starvation arrested after DNA synthesis to produce a population that consisted primarily of 2C unbudded cells (∼98%, *n* = 215) (Fig. 1A). The *in vivo* typical-sized cell population consisted of both 1C and 2C populations; the 1C population was ∼89% unbudded (*n* = 357), while the 2C population was ∼93% unbudded (*n* = 203) (Fig. 1A). Similar to the stationary-phase 2C cells, the *in vivo* typical-sized 2C cell population was primarily unbudded.

Our results show the C. neoformans log-phase cells grown in nutrient-replete media produced a 2C population that was primarily budded, similar to the cell cycle observed in S. cerevisiae, where DNA replication is associated with daughter cell growth and division (Fig. 1B, growth cycle). In contrast, the C. neoformans stationary-phase cells and *in vivo* typical-sized cells produced a 2C population consisting of unbudded cells. These data show C. neoformans cells under both *in vivo* and *in vitro* (nutrient, hypoxia, temperature) (26–29) stress conditions utilize an alternative cell cycle in which DNA replication occurs before any morphological changes associated with daughter cell growth, resulting in an unbudded G_2_ arrest (Fig. 1B, stress cycle).

### Nuclear dynamics are predominantly normal upon cell cycle reentry after unbudded G_2_ arrest.

We envisioned two possible scenarios in response to the unbudded G_2_ arrest. One hypothesis was that the unbudded G_2_ arrest is a cell cycle anomaly and results in an aberrant mitosis upon reentry into the cell cycle. Alternatively, we hypothesized that the unbudded G_2_ arrest is a preprogramed part of the C. neoformans cell cycle. In this scenario, we expected to see little to no disruption in mitosis, with the cells readily adapting to the altered stress cell cycle and 2C (or higher for titan cells) DNA content.

**FIG 2:**
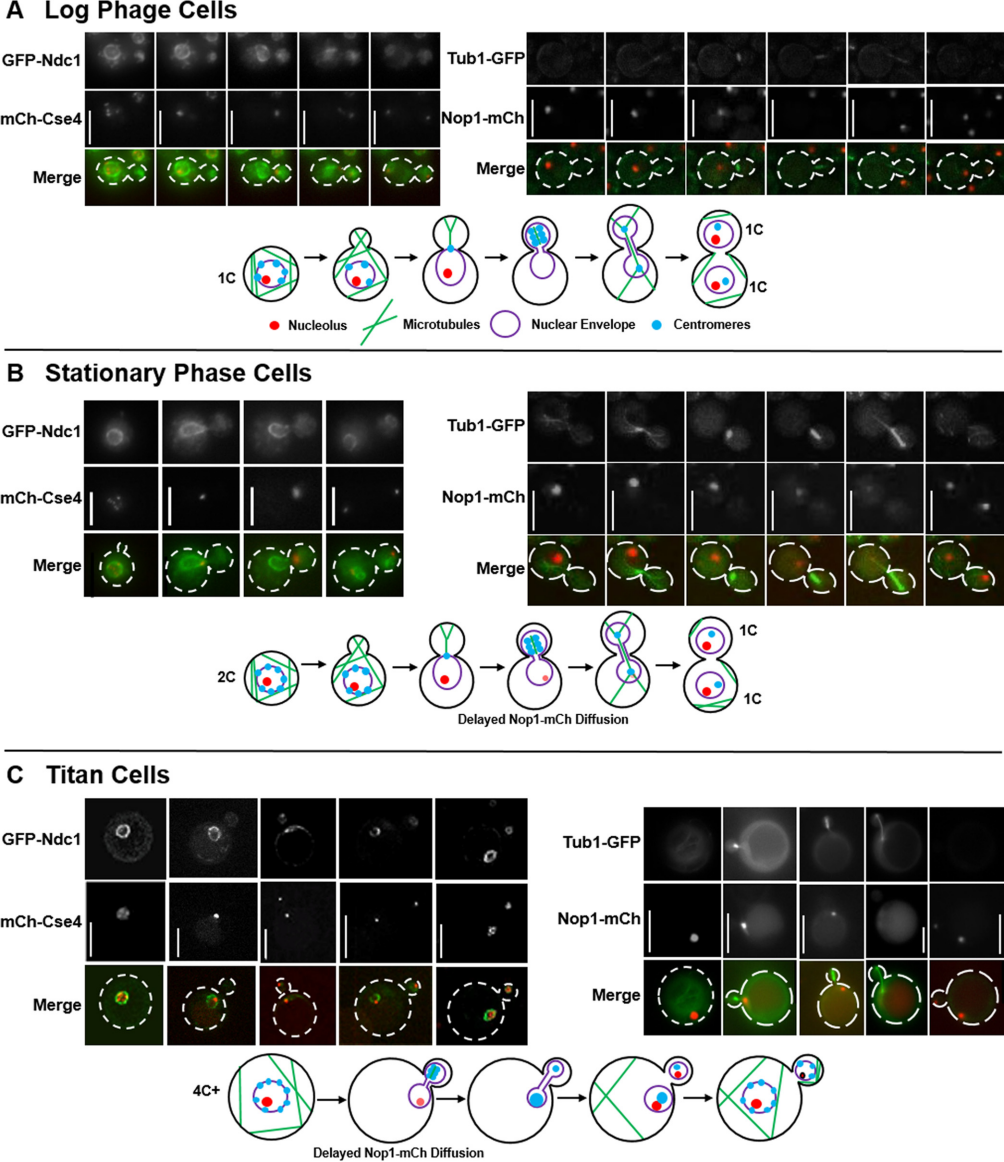
Analysis of nuclear dynamics after unbudded G_2_ arrest shows minimal effect on the ability of stationary and titan cells to complete mitosis and divide. Representative images and corresponding schematic diagrams showing mitotic events in log-phase cells (A), stationary-phase cells (B), and *in vivo* titan cells (C). Centromere and nuclear envelope dynamics were analyzed in cells expressing the inner kinetochore protein, mCherry-Cse4, and the nuclear envelope protein, GFP-Ndc1. Microtubules and nucleolus dynamics were analyzed in cells expressing the nucleolar protein, Nop1-mCherry, and the tubulin protein, Tub1-GFP. All cells were analyzed by time-lapse microscopy, and titan cells were analyzed using both time-lapse and 2-photon microscopy due to their larger size. Not all representative images of the different cell cycle stages are of the same cell. (A) Consistent with previous studies of log-phase cells (25), the centromeres were not clustered in the mother cell prior to mitosis, microtubules formed the mitotic spindle in the daughter cell, and the centromeres then clustered and moved completely into the daughter. Elongation of the spindle resulted in breakage of the nuclear envelope and loss of the nucleolus staining. After mitosis, half of the centromeres returned to the mother cell, and two separate nucleoli and two separate nuclear envelopes were formed, one in the mother and one in the daughter cell. (B) Stationary-phase cells that had already undergone DNA replication prior to bud emergence underwent mitosis similar to log-phase cells. A minor difference in nucleolus retention time was observed. The nucleolus of the stationary-phase cells faded dramatically but was retained throughout the course of mitotic spindle formation and DNA segregation. (C) *In vivo*-derived titan cells also underwent a mitosis similar to the log- and stationary-phase cells, including a slightly delayed disappearance of the nucleolus in the mother cell. The titan cells produced typical-sized daughter cells. Scale bars are 5 μm in panels A and B and 10 μm in panel C.

To test these two hypotheses, we analyzed nuclear dynamics and movement upon cell cycle reentry through analysis of cells expressing fluorescently tagged versions of the proteins Nop1 (nucleolus), Tub1 (tubulin), Cse4 (inner kinetochore), and Ndc1 (nuclear envelope) (Fig. 2). *In vitro* log-phase, *in vitro* stationary-phase, and *in vivo* titan cells were suspended in nutrient replete medium to induce reentry into the cell cycle and analyzed by time-lapse microscopy. Surprisingly, in all cell types only very subtle differences from the log-phase cells were observed. As shown previously, in the log-phase cells the nuclear membrane ruptures, resulting in loss of nucleolus staining as the mitotic spindle elongates into the daughter cell, followed by half of the centromeres translocating back into the mother cell (25) (Fig. 2A). The dynamics of this process were very similar in the *in vitro* stationary phase (Fig. 2B) and *in vivo* titan cells (Fig. 2C). The only observable difference was a delay in loss of nucleolus staining. This was most obvious in the stationary-phase cells where the nucleolus staining diminished dramatically but did not fully disappear (Fig. 2B). A similar phenomenon was observed during titan cell division, where nucleolus signal loss was slightly delayed compared to that of the log-phase cells (Fig. 2C). It should be noted that both the stationary-phase and titan cells are larger than the log-phase cells and have a higher ploidy; thus, the delay in loss of nucleolus staining could be due to either a larger nucleus or cell that results in more time before rupture of the nuclear membrane by the spindle. Taken together, we observed no increased rates of spindle, chromosome localization, or morphological anomalies such as trimeras or elongated buds that would suggest an aberrant mitosis upon reentry into the cell cycle following the unbudded G_2_ arrest, suggesting the unbudded G_2_ arrest was a programmed cell cycle response to stress in C. neoformans.

### The nonessential cyclin Cln1 negatively regulates *in vivo* titan cell formation.

In other human pathogens, DNA replication is linked to cell growth and division, and changes in cell cycle progression affect cell morphology (30–32). Thus, we hypothesized that the unbudded G_2_ arrest and subsequent polyploid titan cell formation is also regulated by the cell cycle.

To explore the role of cell cycle regulation in titan cell formation, we identified 14 putative cyclins and seven cyclin-dependent kinases (CDKs) in the C. neoformans genome based on homology to S. cerevisiae and mammalian cyclins and CDKs (Table 1). Deletion strains were generated in the KN99α genetic background for each of the C. neoformans cyclin and CDK homologs and analyzed for their effect on the *in vitro* cell cycle via ploidy and morphology analysis (Fig. S1A and B). We were unable to obtain deletions for two cyclins (CLB2 and CCL1) and four CDKs (CDK1, SGV1, KIN28, and PHO85), suggesting that these cyclins and CDKs are essential in the KN99α strain. This essentiality was further supported by diploid sporulation assays (see Table S1 in the supplemental material). The expression of *CLB2* and *CCL1* was used to determine when these cyclins likely functioned within the *in vitro* C. neoformans cell cycle (Fig. S1C).

**TABLE 1:** Identification of putative cyclins and cyclin-dependent kinases in C. neoformans

Function^*a*^	Gene ID	Name	Essential^*b*^	Cyclin family
Cyclins	CNAG_06092	Cln1	No	Cdc28
	**CNAG_04575**	**Clb2**	**Yes**	Cdc28
	CNAG_02095	Clb3/Cbc1 (79)	No	Cdc28
	CNAG_00183	Pcl2	No	Pho85
	CNAG_00442	Pcl9	No	Pho85
	CNAG_03385	Pcl103	No	Pho85
	CNAG_02658	Pcl5	No	Pho85
	CNAG_05524	Pcl7	No	Pho85
	CNAG_01922	Pho80	No	Pho85
	CNAG_00024	Clg1	No	Pho85
	CNAG_00440	Ssn801	No	Ssn3
	CNAG_02127	Ssn802	No	Ssn3
	CNAG_05901	Ssn803	No	Ssn3
	**CNAG_04405**	**Ccl1**	**Yes**	Kin28
Cyclin-dependent kinases	**CNAG_01664**	**Cdk1**	**Yes**	
	CNAG_04118	Ctk1	No	
	**CNAG_05549**	**Sgv1**	**Yes**	
	**CNAG_06445**	**Kin28**	**Yes**	
	**CNAG_08022**	**Pho85**	**Yes**	
	CNAG_00415	Cdc2801	No	
	CNAG_06086	Cdk8	No	

a Function of predicted protein product.

b Essential cyclins and CDKs are in boldface and were determined based on diploid sporulation assays (see Table S1 for further detail).

10.1128/mBio.02509-21.1 TABLE S1 Diploid sporulation assay. Download Table S1, PDF file, 0.1 MB. Copyright © 2021 Altamirano et al. 2021 Altamirano et al. https://creativecommons.org/licenses/by/4.0/ This content is distributed under the terms of the Creative Commons Attribution 4.0 International license.

10.1128/mBio.02509-21.4 FIG S1 *In vitro* morphology, ploidy, and expression of cyclin deletion strains. Download FIG S1, PDF file, 0.6 MB. Copyright © 2021 Altamirano et al. 2021 Altamirano et al. https://creativecommons.org/licenses/by/4.0/ This content is distributed under the terms of the Creative Commons Attribution 4.0 International license.

To assess titan cell formation in each of the deletion strains, we infected mice with the deletion strain or the corresponding complement strain and assessed the ability of each to produce titan cells (Fig. S2). The *clb3Δ* and *ssn803Δ* mutants had severe 37°C growth defects, and cells from mice were insufficiently recovered to analyze their titan cell formation (data not shown and Fig. S2). Deletion of the cyclins Pcl5, Clg1, Ssn802, and the CDK Ctk1 had subtle effects on titan cell formation that were rescued by complementation.

10.1128/mBio.02509-21.5 FIG S2 *In vivo* titan formation in cyclin and CDK deletion strains. Download FIG S2, PDF file, 0.2 MB. Copyright © 2021 Altamirano et al. 2021 Altamirano et al. https://creativecommons.org/licenses/by/4.0/ This content is distributed under the terms of the Creative Commons Attribution 4.0 International license.

**FIG 3:**
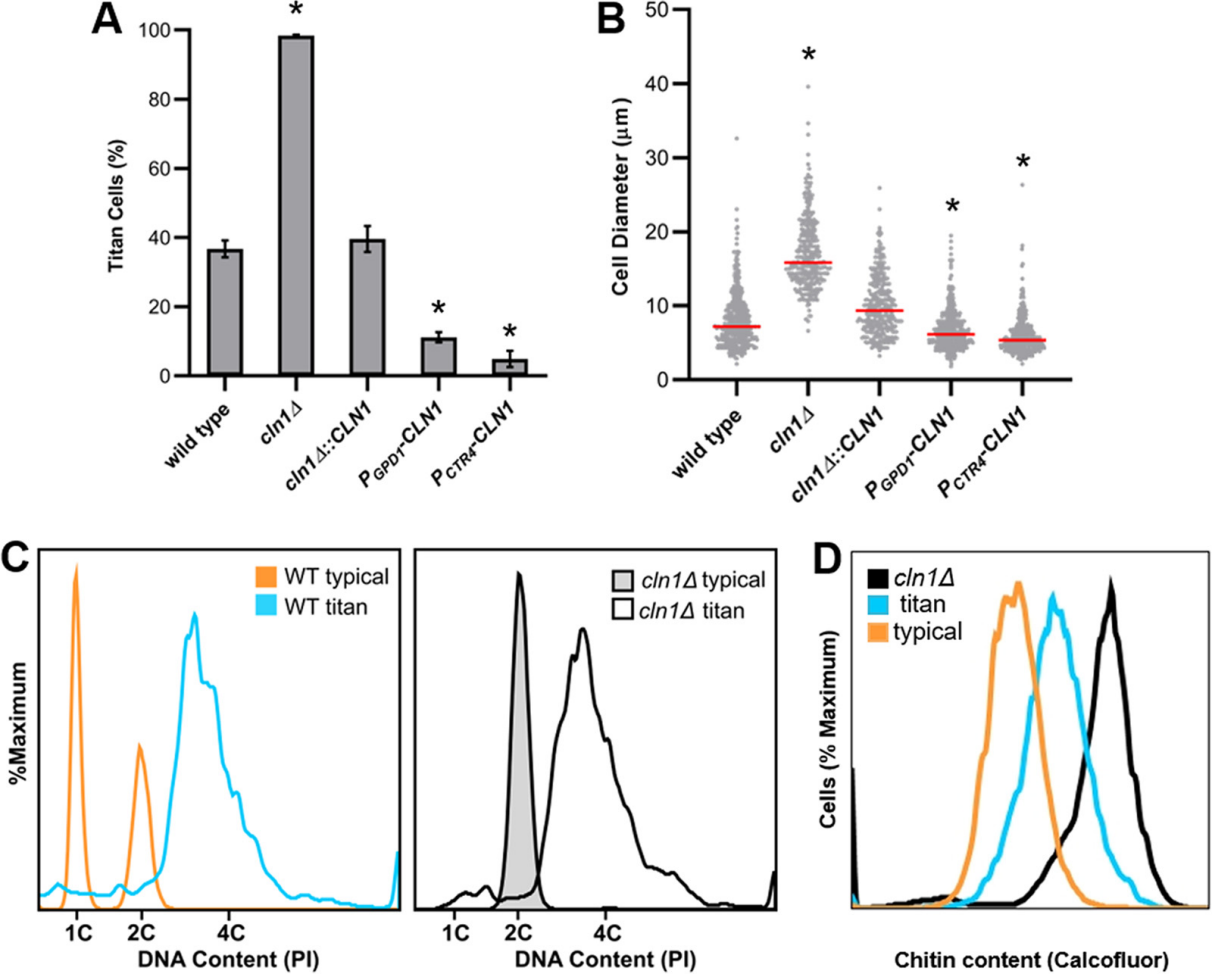
Cln1 negatively regulates *in vivo* titan cell formation. Mice were infected via inhalation with 5 × 10^4^ cells of the wild-type strain (KN99α), the *cln1Δ* deletion, the *cln1Δ*::*CLN1* complement, or the overexpression strains *P_GPD1_-CLN1* or *P_CTR4_-CLN1*. Titan cell formation in the lungs was analyzed at 3 days postinfection. (A) Percentage of titan cells was determined based on a cell body size threshold of 10 μm, excluding the capsule, for >300 cells per mouse. Error bars indicate standard deviations (SD), *n* ≥ 3 mice per strain. *, *P* < 0.05 compared to the wild type by Student's *t* test with Welch’s correction. (B) Cell body size, excluding the capsule, was also plotted for >300 cells per mouse as a visual representation of the distribution of cell size in the different strains. Median cell size is indicated by the red line. *, *P* < 0.05 compared to wild type by Kruskal-Wallis with Dunn posttest. (C) *cln1Δ* cells isolated from the lungs of mice were fixed, stained with propidium iodide, and analyzed by flow cytometry for DNA content to determine cell ploidies within the population. Wild-type *in vitro*-grown cells (orange), wild-type titan cells (blue), *in vitro*-grown *cln1Δ* cells (gray), and a diploid strain (not shown) were used as controls. (D) Wild-type and *cln1Δ* cells isolated from the lungs of mice were fixed, stained with calcofluor white, and analyzed by flow cytometry to determine cell wall chitin content. The wild-type population was split based on cell size into the typical (orange) and titan (blue) cell subsets for chitin analysis.

Interestingly, deletion of the cyclin gene, *CLN1*, led to 98% ± 0.27% titan cell formation (Fig. 3A) (*P* = 0.0000016), while the complement exhibited titan cell production similar to that of the wild type. To further investigate the role of C. neoformans Cln1 in titan cell production, we generated two *CLN1* overexpression strains, one overexpression strain with *CLN1* under the control of the constitutive promoter, *GPD1* (33), and a second overexpression strain with *CLN1* under the control of the copper repressible promoter, *CTR4*. The *CTR4* promoter is turned on in the absence of copper and turned off in the presence of copper (34). The lung environment is a copper-limiting environment (35), resulting in overexpression of *CLN1* in the *P_CTR4_-CLN1* strain *in vivo*. *CLN1* overexpression reduced titan cell production compared to the wild-type strain (Fig. 3A) (*P* =  0.000011). The *cln1Δ* cells had a significant increase in cell body diameter compared to the wild-type cells, while the overexpression strains had cell body sizes smaller than the wild-type cells (Fig. 3B). In addition to their large size, titan cells are defined by their increased ploidy and the high chitin content in their cell wall (36–38). The large *cln1Δ* cells isolated from mice had higher levels of both DNA and chitin, indicating the *cln1Δ* cells are bona fide titan cells (Fig. 3C and D). Overall, these data show *CLN1* gene expression negatively regulates titan cell formation during *in vivo* infection.

### Release from stationary phase into nutrient-replete media induces morphological defects in the *cln1Δ* mutant.

We showed Cln1 negatively regulates titan cell formation during infection, resulting in a dramatic increase in titan cell formation, and that the lung environment triggers an unbudded G_2_ arrest. Our preliminary *in vitro* studies of the *cln1Δ* deletion strain revealed only minor defects in log-phase growth in nutrient-replete media compared to wild-type cells. Overnight cultures of the *cln1Δ* deletion strain exhibited a slight increase in overall cell size, were primarily unbudded 2C cells, and exhibited a more severe growth defect at 37°C than wild-type cells (Fig. S1 and S3). These results led us to hypothesize that Cln1 regulates the unbudded G_2_ arrest. To test this hypothesis, we took advantage of our ability to produce an unbudded 2C population through nutrient starvation *in vitro* and evaluated the capacity of *cln1Δ* cells to enter and exit stationary phase.

10.1128/mBio.02509-21.6 FIG S3 The *cln1*Δ deletion strain has a growth defect at 37°C. Download FIG S3, PDF file, 0.3 MB. Copyright © 2021 Altamirano et al. 2021 Altamirano et al. https://creativecommons.org/licenses/by/4.0/ This content is distributed under the terms of the Creative Commons Attribution 4.0 International license.

**FIG 4:**
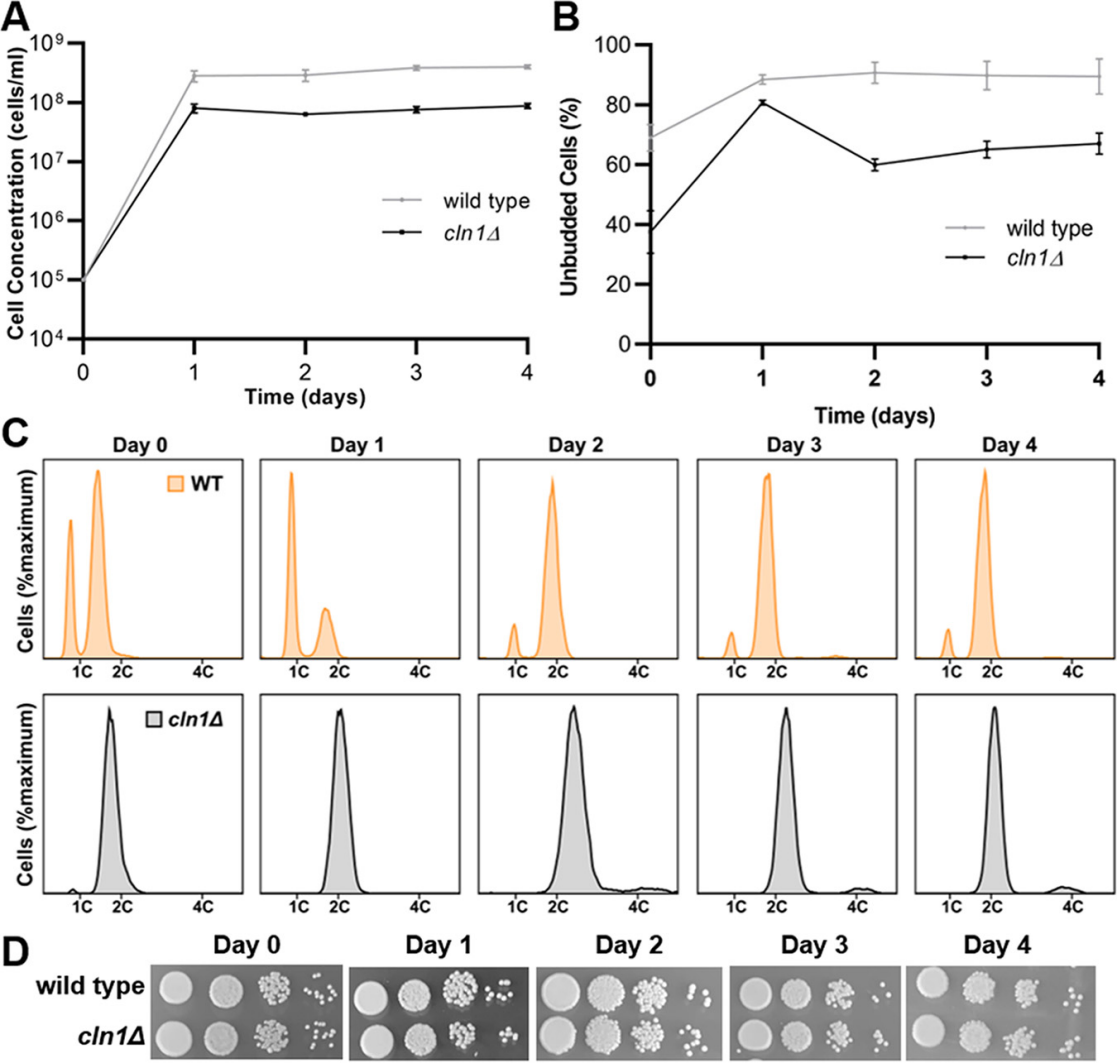
*cln1Δ* cells arrest as 2C cells and maintain viability during nutrient deprivation. Wild-type and *cln1Δ* cells were grown in YPD liquid medium for 4 days and assessed for entry into stationary phase at various time points based on cell concentration, morphology, viability, and ploidy. Error bars represent SD from three biological replicates. (A) Wild-type (gray) and *cln1Δ* (black) cells displayed a plateau in cell growth after 1 day of culture. (B) By 1 day, when nutrients became limited, both wild-type and *cln1Δ* cells were primarily unbudded, while the *cln1Δ* cells contained more budded cells at the later time points. (C) DNA content analysis showed both strains arrested as 2C cells. The *cln1Δ* cells were primarily 2C throughout the experiment, while the wild-type cells arrested as 2C cells only after 2 days in YPD liquid media. (D) Spot assays were performed at days 0, 1, 2, 3, and 4 of nutrient deprivation, and no differences in viability were observed.

The ability to enter stationary phase was assessed based on cell concentration, morphology, ploidy, and viability. The *cln1Δ* deletion strain entered stationary phase at a slightly lower cell concentration than the wild type but was otherwise able to maintain an appropriate stationary population (Fig. 4A). While the majority of the wild-type cells remained unbudded throughout nutrient deprivation, buds were observed starting at day 2 in roughly 40% of the *cln1Δ* cells (Fig. 4B). Cells in the *cln1Δ* strain had 2C ploidy under nutrient starvation at an earlier time point than the wild type, although it should be noted that more of the *cln1Δ* cells are in 2C in the log-phase cultures at the start of the experiment (Fig. 4C). Coincident with new bud formation on day 2, the *cln1Δ* deletion also developed a small population of 4C cells starting on day 2. Finally, no difference in viability was observed between the *cln1Δ* and wild-type cells when tested using spot assays on YPD medium (Fig. 4D). These data suggest Cln1 is not required for the unbudded G_2_ stationary-phase arrest or viability but may be involved in maintaining control over cell morphology and ploidy during the unbudded G_2_ arrest.

**FIG 5:**
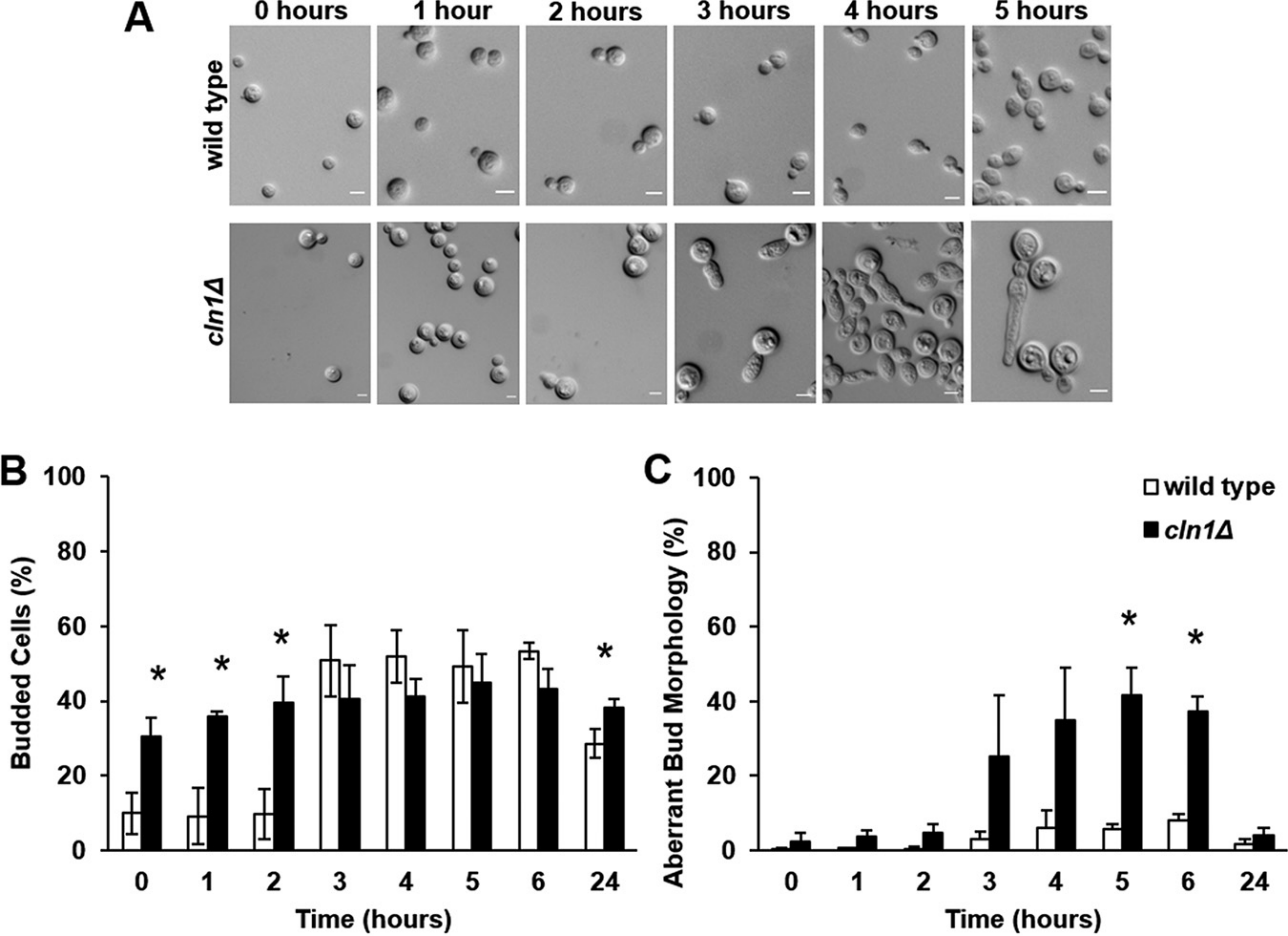
*cln1Δ* cells exhibit a transient aberrant budding morphology after release from nutrient deprivation. After 3 days of incubation under nutrient starvation conditions, wild-type and *cln1Δ* cells were pelleted and resuspended into fresh YPD liquid medium. Error bars represent SD from three biological replicates. (A) The *cln1Δ* cells exhibited an elongated bud morphological defect and delayed bud formation. Scale bar, 5 μm. (B) While the wild-type cells initiated budding at 3 h posttransfer, no increase in *cln1Δ* cell budding was observed posttransfer. (C) The *cln1Δ* cells had a transient aberrant bud morphology that peaked at 5 h posttransfer but was not observed at 24 h. *, *P* < 0.05 by Student's *t* test with Welch’s correction.

To more thoroughly assess cell morphology after stationary-phase release, we released cells into liquid media and evaluated the cell morphology of each strain at various time points (Fig. 5). After release, the *cln1Δ* cells exhibited a delay in budding and an aberrant bud morphology (Fig. 5A). By 3 h after release, *cln1Δ* cells had an elongated bud morphology, resulting in pseudohypha-like cells, with some daughter cells initiating another bud site without undergoing cytokinesis (Fig. 5A). This aberrant bud morphology peaked at 5 h after release in the *cln1Δ* mutant and was transient; by 24 h after release, the *cln1Δ* cells consisted primarily of budded/unbudded cells, containing only a minority of cells with an aberrant morphology that was not significantly different from the wild-type culture (*P* = 0.163743) (Fig. 5B and C). These data show Cln1 is important for regulating morphological changes associated with reentry into the cell cycle following the stationary-phase unbudded G_2_ arrest.

### Cln1 binding alters Cdk1 kinase activity.

**FIG 6:**
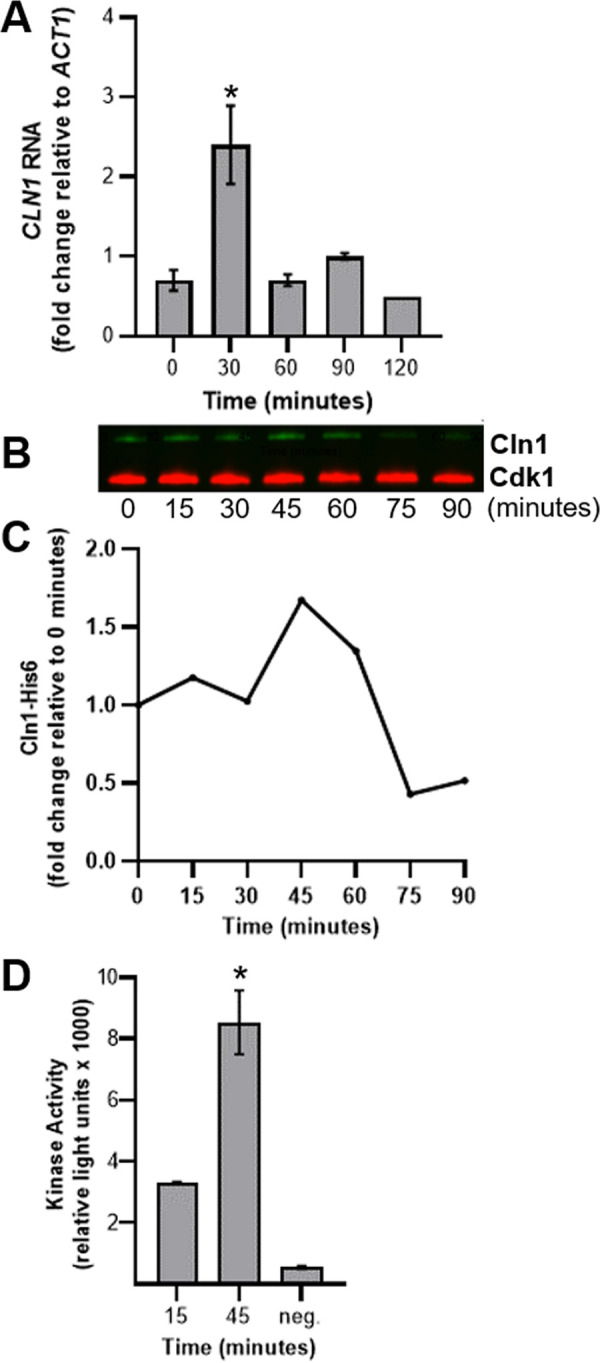
Cln1 activates Cdk1 to initiate bud formation. (A) CLN1 RNA levels in wild-type cells were analyzed by RT-qPCR at 30-min intervals after release from stationary phase into nutrient-replete media. Expression levels were normalized to the ACT1 housekeeping gene to determine changes in gene expression. Error bars represent SD of the *C_T_* values from 3 technical replicates. Data are representative of 3 biological replicates. *, *P* < 0.05 compared to *t* = 0 by Student's *t* test with Welch’s correction. (B and C) Cells were harvested at 15-min intervals, and the amount of Cln1-His6 coprecipitated with Cdk1-Myc was determined at each time point by Western blotting (B) and then quantified based on the 0-min time point (C). (D) Cdk1 kinase activity was determined at 15 and 45 min. The negative control consisted of the 1× kinase buffer only. Error bars represent SD from 3 technical replicates. *, *P* < 0.05 compared to *t* = 15 by Student's *t* test.

We also measured the expression of *CLN1* in wild-type cells after stationary-phase release and the impact of Cln1 protein binding on activity of cyclin-dependent kinase 1, Cdk1, the primary cyclin-dependent kinase regulating cell cycle progression in C. neoformans (39). Expression profiling by reverse transcription-quantitative PCR (RT-qPCR) showed *CLN1* expression peaks 30 min after release from stationary phase (Fig. 6A). In this analysis, the cells released from stationary phase initiated bud formation 30 min after release, with peak budding occurring by 60 min after release (data not shown). To monitor interactions between Cln1 and Cdk1, we generated fusion proteins using His and Myc tags, respectively (Fig. S4). Consistent with the expression data, peak interaction of Cln1-His with Cdk1-Myc occurred between 30 and 60 min after release from stationary phase (Fig. 6B and C). This interaction increased the Cdk1 kinase activity (Fig. 6D). Combined, these data show that peak *CLN1* expression results in Cln1 binding to Cdk1, increasing the Cdk1 kinase activity that ultimately results in initiation of bud formation.

10.1128/mBio.02509-21.7 FIG S4 The Cln1 cyclin interacts with the cyclin-dependent kinase CDK1. Download FIG S4, PDF file, 0.09 MB. Copyright © 2021 Altamirano et al. 2021 Altamirano et al. https://creativecommons.org/licenses/by/4.0/ This content is distributed under the terms of the Creative Commons Attribution 4.0 International license.

### C. neoformans Cln1 contains both S. cerevisiae Cln and Clb cyclin motifs.

**FIG 7:**
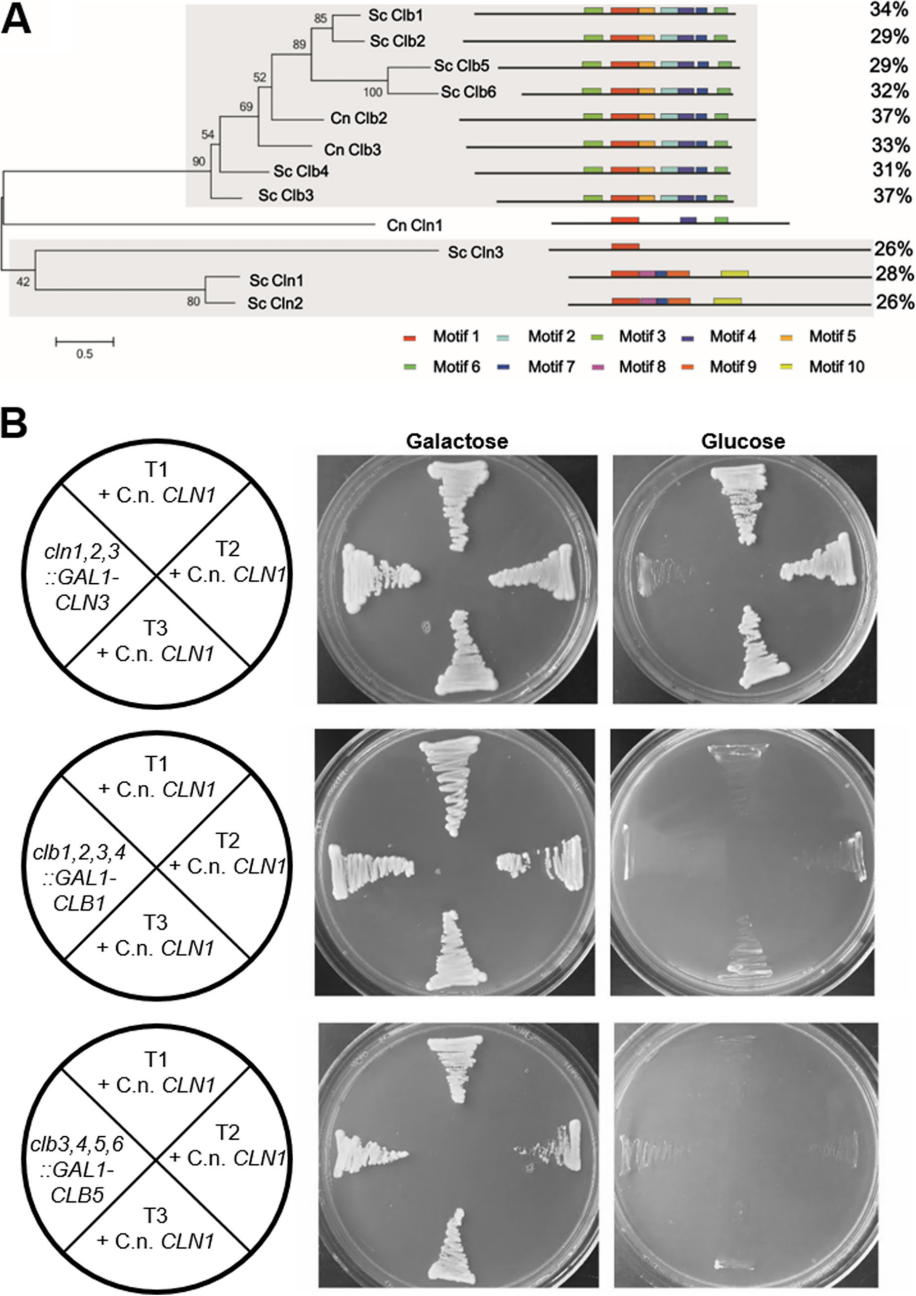
Motif analysis of C. neoformans Cln1 shows it contains both S. cerevisiae Cln and Clb motifs but only complements Cln cyclins in S. cerevisiae. (A) Phylogenetic analysis based on protein motifs of the Cdc28 family of cyclins from C. neoformans (Cln1, Clb2, and Clb3) and S. cerevisiae (Cln1, Cln2, Cln3, Clb1, Clb2, Clb3, Clb4, Clb5, and Clb6) showed C. neoformans Cln1 had highest homology to the S. cerevisiae Clb proteins. (B) C. neoformans *CLN1* expressed in S. cerevisiae was able to rescue growth of a *cln1*,*2*,*3* triple but not *clb1*,*2*,*3*,*4* or *clb3*,*4*,*5*,*6* quadruple mutants. C. neoformans *CLN1* was expressed with a constitutive S. cerevisiae promoter, and all *cln1*,*2*,*3*::*GAL-CLN3 *+* *C. neoformans *CLN1* transformants (T1, T2, or T3 + C.n. *CLN1*) produced growth on the nonpermissive glucose medium, whereas none of the *clb1*,*2*,*3*,*4*::*GAL1-CLB1 *+* *C. neoformans *CLN1* or *clb3*,*4*,*5*,*6*::*GAL-CLB5 *+* *C. neoformans *CLN1* transformants had growth on the nonpermissive glucose medium.

C. neoformans Cln1 is a homolog of the S. cerevisiae Cdc28 cyclins (Table 1). In S. cerevisiae, there are nine Cdc28 cyclins: the G_1_ cyclin Cln3; two G_1_/S transition cyclins, Cln1 and Cln2; two S cyclins, Clb5 and Clb6; two G_2_/M cyclins, Clb3 and Clb4; and two M cyclins, Clb1 and Clb2. In contrast, the Cryptococcus species have only three Cdc28 cyclin homologs. Additionally, although both C. neoformans and S. cerevisiae divide by budding, there are key differences in their cell cycles (23, 25) (Fig. 1 and 2). To investigate the evolutionary relationship between C. neoformans Cln1 and the S. cerevisiae Cdc28 cyclins, we performed a phylogenetic analysis and compared the conserved motifs among the Cdc28 cyclins from both C. neoformans and S. cerevisiae (Fig. 7A, Fig. S5). Interestingly, C. neoformans Cln1 exhibited similarity with both S. cerevisiae Cln and Clb cyclins and had highest motif similarity to S. cerevisiae Clb3 (Fig. 7A).

10.1128/mBio.02509-21.8 FIG S5 Conserved motif sequences of the Cdc28 cyclins from C. neoformans and S. cerevisiae. Download FIG S5, PDF file, 0.3 MB. Copyright © 2021 Altamirano et al. 2021 Altamirano et al. https://creativecommons.org/licenses/by/4.0/ This content is distributed under the terms of the Creative Commons Attribution 4.0 International license.

The *C. deneoformans CLN1* gene was previously shown to complement the S. cerevisiae Cln cyclins (40) but was not tested for Clb cyclin complementation. In C. neoformans, peak *CLN1* expression occurs after release from stationary phase at the G_2_-to-M transition (Fig. S1). In addition, the elongated bud morphology observed in the *cln1Δ* cells is similar to the elongated bud phenotype observed in S. cerevisiae cells deficient in the mitotic cyclin Clb2 that regulates the G_2_-to-M cell cycle transition (41, 42). These data led us to hypothesize that the Cln1 cyclin in C. neoformans acts at the G_2_-to-M transition in the unbudded G_2_ arrest stress cell cycle and, given the motif similarity, may be able to also complement the S. cerevisiae Clb cyclins.

To test this hypothesis, we analyzed whether C. neoformans Cln1 can functionally complement the S. cerevisiae Cln and Clb cyclins. The S. cerevisiae *cln1Δ cln2Δ cln3Δ* triple mutant, when complemented with *CLN3* fused to a galactose-inducible promoter, is deficient for Cln cyclins in the presence of glucose, resulting in no growth. In contrast, the triple mutant strain grows on galactose where *GAL-CLN3* is expressed. Similar to *C. deneoformans* (40), addition of the C. neoformans *CLN1* gene-coding region under the control of the S. cerevisiae *GPD1* constitutive promoter restored growth of the S. cerevisiae *cln1Δ cln2Δ cln3Δ GAL-CLN3* cells on glucose, showing that C. neoformans Cln1 can functionally complement the S. cerevisiae Cln cyclins (Fig. 7B). When we performed the same complementation experiment with the Clb-deficient strain *clb1Δ clb2Δ clb3Δ clb4Δ GAL-CLB1* or *clb3Δ clb4Δ clb5Δ clb6Δ GAL-CLB5*, C. neoformans Cln1 was unable to functionally complement the S. cerevisiae Clb cyclins (Fig. 7B). Taken together, these comparative studies between the S. cerevisiae cyclins and C. neoformans Cln1 showed that Cln1 has homology to both the Cln and Clb cyclins in S. cerevisiae but only functionally complements the S. cerevisiae Cln cyclins.

### Low *CLN1* expression after unbudded G_2_ arrest leads to polyploid titan cell formation.

The functional complementation experiments in S. cerevisiae identified a possible role for C. neoformans Cln1 only in regulating early phases of the cell cycle that are controlled by Cln cyclins in S. cerevisiae, yet our observations that C. neoformans *CLN1* expression peaked upon initiation of budding after release from stationary-phase growth arrest, the elongated bud morphology observed in the *cln1Δ* deletion upon release from stationary-phase *in vitro*, the homology to the S. cerevisiae Clb cyclins, and activation of the C. neoformans cyclin-dependent kinase Cdk1 by Cln1 at bud formation all indicated a role for C. neoformans Cln1 in later phases of the cell cycle. Given the further observation that C. neoformans has a stress-induced unbudded G_2_ arrest, we hypothesized that Cln1 is critical for maintaining the balance between cell growth and DNA replication during this unbudded G_2_ arrest.

**FIG 8:**
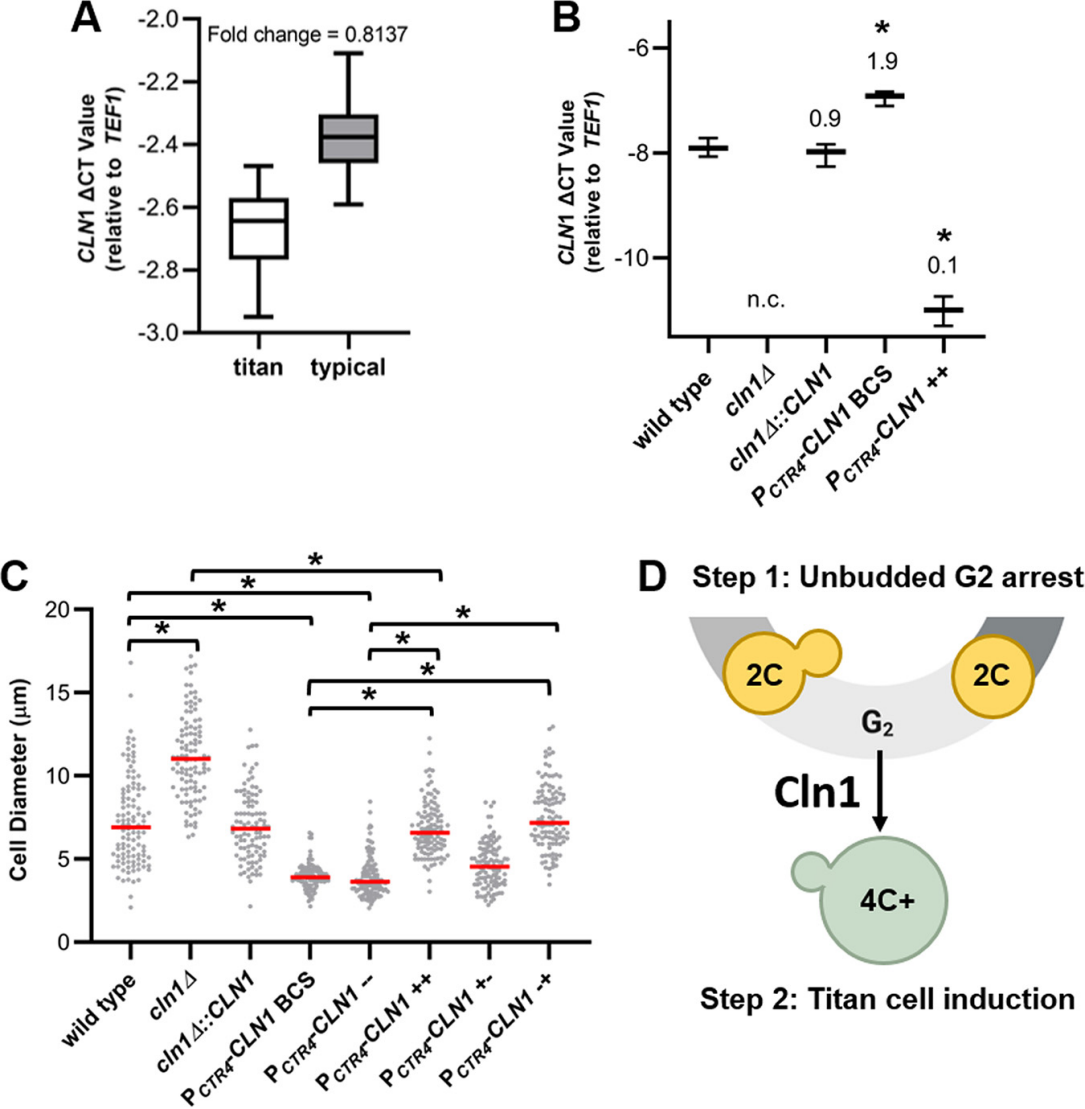
Low *CLN1* expression after unbudded G_2_ arrest induces titan cell formation. (A) RT-qPCR showed higher *CLN1* levels in typical-size cells than titan cells isolated from the lungs of mice. Expression levels were normalized to both cell number and the TEF1 housekeeping gene to determine change in cycle threshold (Δ*C_T_*). Fold decrease in gene expression in titan compared to typical cells was calculated using 2-ΔΔ*C_T_*. Data presented are the Δ*C_T_* values from three biological replicates. *P* = 0.0004 by Student's *t* test. (B) RT-qPCR analysis of CLN1 expression *in vitro* in the wild-type, *cln1Δ*, and *cln1Δ*::*CLN1* strains and when the copper-repressible promoter strain was grown with 400 μM of the copper chelator BCS (*P_CTR4_-CLN1* BCS) or 25 μM copper (P_CTR4_-CLN1++). No expression change was detected in the *cln1Δ* strain (n.c). Fold change compared to the wild type is indicated above each bar. Data presented are the Δ*C_T_* values from three technical replicates. *, *P* ≤ 0.0015 compared to the wild type by one-way ANOVA with a Dunnett correction for multiple comparisons. (C) *In vitro* titan cell formation with wild-type, *cln1Δ*, *cln1Δ*::*CLN1*, and *P_CTR4_-CLN1* cells showed *CLN1* expression only affects titan cell formation after unbudded 2C cells are already formed. *In vitro* titan cell formation was induced using an initial stationary-phase culture to induce unbudded G_2_ arrest followed by a second incubation under hypoxic conditions to induce titan cell formation (43). *CLN1* expression was manipulated using the *P_CTR4_-CLN1* strain and copper addition during the initial stationary culture (+−), second incubation (−+), both (++), or neither (−−). The cell diameter of at least 100 cells was measured at the end of the protocol to determine *in vitro* titan cell formation. Median cell size is indicated by the red line. *, *P* < 0.0001 by Kruskal-Wallis test with Dunn’s posttest correction. (D) Our results show Cln1 is necessary in the stress cell cycle after the unbudded 2C cells are already formed. Loss or reduction of *CLN1* expression in cells after unbudded G_2_ arrest results in polyploid titan cells (green cells) only with concomitant environmental stimuli.

We first explored the association between *CLN1* expression levels in titan and typical-sized cells *in vivo*, testing the hypothesis that low Cln1 levels would be unable to maintain control of the unbudded G_2_ arrest and result in titan cell formation. RT-qPCR analysis of *CLN1* RNA isolated from typical and titan cells and normalized to cell number showed lower *CLN1* expression in titan cells than typical-sized cells *in vivo* (Fig. 8A) (*P* =  0.0004). While these results provide indirect support for our hypothesis that low levels of Cln1 in a cell would be unable to maintain the unbudded G_2_ arrest and lead to titan cell formation, we were unable to adequately manipulate the *in vivo* system.

Methods to generate titan-like cells *in vitro* were recently identified (43–45), allowing us to manipulate *CLN1* expression (Fig. 8B) and monitor the effect on titan cell formation. Furthermore, the *in vitro* titan cell assays require a two-step process to induce titan cell formation. The first step induces the unbudded G_2_ arrest, and the second step induces titan cell formation using environmental cues. By manipulating *CLN1* expression during these two steps, we can pinpoint whether Cln1 is critical to maintaining the unbudded G_2_ arrest or to control titan cell formation or is acting at both steps in the process of titan cell formation.

As previously shown for *in vivo* titan cell formation, the *cln1Δ* deletion strain had increased *in vitro* titan cell formation compared to the wild-type strain (*P* < 0.0001) and no *CLN1* gene expression (Fig. 8B and C). The *P_CTR4_-CLN1* strain had decreased titan cell formation compared to the wild-type strain in the *in vitro* assay, both with and without depletion of copper in the media using bathocuproinedisulfonic acid (BCS) (*P* < 0.0001), that resulted in an almost 2-fold increase in *CLN1* expression compared to the wild type (Fig. 8B and C). When copper was present in the media (*P_CTR4_-CLN1*++), the *CLN1* expression in the *P_CTR4_-CLN1* strain decreased significantly (fold change in expression, 0.1) and resulted in titan cell formation similar to that of the wild-type strain (*P* > 0.9999) but lower than the *cln1Δ* deletion strain (*P* < 0.0001) (Fig. 8B and C). The observation that the *P_CTR4_-CLN1++* strain in the presence of copper does not exhibit the same increase in titan cell formation as the *cln1Δ* deletion strain has two possible explanations. The most likely explanation is that the copper-repressible promoter is leaky and there is still a low level of *CLN1* expression. Consistent with this explanation, the P_CTR4_-CLN1 strain in the presence of copper does not display complete abolishment of *CLN1* expression (Fig. 8B). A second explanation is that some of the signals leading to titan cell formation are present in the *CLN1* promoter, and replacement of the *CLN1* promoter with the *CTR4* promoter produced a strain unable to overproduce titan cells when *CLN1* is overexpressed.

The conditions we used for *in vitro* titan cell formation required an initial first step that induced the unbudded G_2_ arrest using nutrient starvation prior to shifting the cells to titan-inducing conditions in the second step. Thus, we dissected the role of Cln1 in titan cell formation using the *P_CTR4_-CLN1* strain both in the initial incubation that results in the G_2_ arrest and the subsequent incubation that produces the titan cells (Fig. 8C). When we repressed *CLN1* only in the initial incubation (*P_CTR4_-CLN1*−+) to test if Cln1 was required during the G_2_ arrest, we observed no differences in titan cell formation compared to the *P_CTR4_-CLN1 BSC* and *P_CTR4_-CLN1−−* controls (*P* > 0.9999). In contrast, when *CLN1* was repressed in the second incubation (*P_CTR4_-CLN1+−*), titan cell formation increased compared to the *P_CTR4_-CLN1 BSC* and *P_CTR4_-CLN1−−* controls (*P* < 0.0001). These results show Cln1 activity is only necessary after the unbudded G_2_ arrest. Under appropriate environmental conditions, insufficient *CLN1* expression results in polyploid titan cell formation following the unbudded G_2_ arrest (Fig. 8D).

## DISCUSSION

Although titan cells play a role in virulence in the pathogenic yeast C. neoformans, the mechanisms underlying titan cell formation are still largely unknown. Previous studies exploring the host signals and signal transduction pathways stimulating titan cell formation showed their production is tightly regulated and has similarities to morphological switches in other fungal species that are cell cycle regulated (43, 46–50). We characterized the cell cycle of C. neoformans during *in vivo* and *in vitro* stress to better understand potential mechanisms leading to titan cell formation and linked these studies to identification of the putative cyclins and CDKs to investigate their roles in titan cell formation. Our results show that titan cell formation is cell cycle regulated, and the cyclin Cln1 negatively regulates titan cell formation. Additionally, we show that C. neoformans produces unbudded G_2_ cells in response to *in vivo* and *in vitro* stresses and that Cln1 is critical for regulation of morphogenesis after this G_2_ arrest, leading us to postulate that the unbudded 2C cell is a precursor to titan cells (Fig. 8D).

Previous studies identified Cln1 in Cryptococcus, but the link to titan cell formation, and its exact role in the cell cycle, remained unclear (40, 51, 52). To better understand these mechanisms and the role of Cln1 in the unbudded G_2_ arrest, we assessed the ability of C. neoformans Cln1 to functionally complement Cln and Clb cyclins in S. cerevisiae. Cln1 in the closely related species *C. deneoformans* had already been shown to complement the S. cerevisiae Cln cyclins (40), so we were unsurprised that the C. neoformans Cln1 was also able to complement the Cln cyclins. Additionally, we showed that C. neoformans Cln1 does not functionally complement the S. cerevisiae Clb cyclins. This result may seem contradictory to our data showing that C. neoformans Cln1 is involved in cell cycle regulation after the unbudded G_2_ arrest. However, there are a number of important differences between the S. cerevisiae and C. neoformans cell cycles that may shed light on the unique functionality of Cln1 in C. neoformans.

First, in S. cerevisiae, the Cln cyclins are associated with the initiation of START, a cell cycle commitment point that occurs at the G_1_-S transition. The presence of START in S. cerevisiae ensures budding and DNA replication are tightly coordinated (53). In contrast, our cell-sorting studies in C. neoformans show that DNA replication without bud formation readily occurs both *in vivo* and *in vitro* and are consistent with previous microscopy studies in both C. neoformans and *C. deneoformans* (18, 26–29, 54). These data suggest that START does not exist in Cryptococcus. This lack of START likely allows Cln1 to be multifunctional across the C. neoformans cell cycle.

An alternate possibility is that C. neoformans Cln1 has novel functions in the later part of the cell cycle due to its homology to the Clb cyclins. The elongated bud morphology observed in the *cln1Δ* cells is similar to the elongated bud phenotype observed in S. cerevisiae cells deficient in the mitotic cyclin Clb2 that regulates the G_2_-to-M cell cycle transition and inhibits polarized bud growth to initiate the apical-to-isotropic switch that allows the cell to maintain a normal bud morphology. Clb2-deficient cells in S. cerevisiae exhibit a G_2_ mitotic delay, characterized by elongated hyperpolarized buds (41, 55, 56). It should be noted, however, that the elongated bud phenotype is only transiently observed in the C. neoformans *cln1Δ* mutant *in vitro* upon release from stationary phase, and a similar elongated bud phenotype is also seen in S. cerevisiae when *CLN1* is expressed during G_2_ (41).

Finally, the C. neoformans cell cycle shares more similarities to that of mammals and other multicellular eukaryotes than the other model yeasts, such as S. cerevisiae and S. pombe. For example, C. neoformans undergoes a stepwise kinetochore assembly (25) and contains several microtubule-organizing centers (MTOCs) (57). In addition, while S. cerevisiae and S. pombe have a closed mitosis (58–60), C. neoformans has a semiopen mitosis (25) that is more similar to the open mitosis observed in mammals. In addition, many multicellular eukaryotes, including humans, also have cell cycles that do not rely heavily on START; this cell cycle commitment checkpoint is often referred to as the “restriction point” in higher eukaryotes. Specifically, cancer cells are well known for their ability to bypass the restriction point, often resulting in polyploidy (61).

Our studies show that *CLN1* expression increases upon release from stationary phase and is also lower in titan cells than typical-sized cells *in vivo*. During log-phase growth, C. neoformans utilizes a cell cycle characterized by synchronous DNA replication and bud formation that is also observed in S. cerevisiae (25). During stationary phase and *in vivo* growth, we observed a pronounced G_2_ arrest. This unbudded G_2_ arrest was observed previously in various *C*. *deneoformans* and C. neoformans strains in response to other *in vitro* stresses in addition to nutrient deprivation (26, 27), including high temperature (29) and hypoxia (28); thus, we refer to this alternative cell cycle as the Cryptococcus “stress cell cycle.” Our *in vitro* stationary phase and titan cell formation experiments with the *P_CTR4_-CLN1* strain are important for several reasons. First, the stationary-phase studies show Cln1 has minimal effect on entry into G_2_ arrest, corroborated by the titan cell formation experiments. Second, because *in vitro* titan cell formation utilizes a two-step process, we can pinpoint where Cln1 acts in the cell cycle to regulate titan cell formation. Our studies revealed that Cln1 functions after the G_2_ arrest, at the critical decision point when the cell either remains arrested, undergoes DNA rereplication to form a titan cell, or reenters the cell cycle to produce a daughter cell.

In addition, the *in vitro* titan cell formation experiments highlight the fact that while titan cell formation is affected by low Cln1 levels in the cell, low Cln1 is not the only mechanism underlying titan cell formation. Under conditions that do not promote titan cell production, such as *in vitro* log- and stationary-phase growth, the *cln1Δ* mutant does not produce titan cells. These data show C. neoformans needs additional signals to produce titan cells and are consistent with previous studies showing that multiple signal transduction pathways affect titan cell morphogenesis and that production of titan cells *in vitro* requires complex environmental conditions that involve multiple forms of induction (37). There are a variety of signals that affect titan cell formation, but our data highlight the need for the unbudded 2C cell as one of the earliest of these required signals.

The mechanism by which low Cln1 levels in the cell lead to titan cell formation still remains unclear. We postulate that low Cln1 levels in the cell result in DNA replication in the absence of cell division, or DNA rereplication, under environmental conditions that stimulate isotropic growth of the cell, ultimately producing the titan cell phenotype (Fig. 8D). This DNA rereplication may result from premature mitotic exit, a process referred to as mitotic slippage. Mitotic slippage is known to form polyploid cancer cells (62, 63). Alternatively, Cln1-deficient cells may not arrest in G_2_ but instead continue to undergo mitosis, resulting in nuclear division in the absence of cell division, possibly leading to polyploidy through mitotic collapse such as that previously described in C. albicans (64). In C. albicans, mitotic collapse is associated with the production of a trimera cell morphology. In our studies we did not observe trimeras or evidence of mitotic spindle collapse in our time lapse microscopy (S. Altamirano, M. S. Fu, and K. Nielsen, unpublished data). However, in C. neoformans the lack of START and the unbudded G_2_ arrest may make the processes of mitotic slippage and mitotic collapse morphologically indistinguishable.

Cell cycle arrest is an important stress response mechanism in many organisms (65, 66). Both our *in vivo* studies and the previous *in vitro* studies with nutrient, hypoxia, and temperature stress identified the production of 2C unbudded cells in response to various stresses (26–29, 54). Why might 2C arrest be beneficial under stressful conditions? Cell cycle arrest could promote changes in cell morphology or development that allows for increased adaptation to external stress signals. For example, Fu et al. previously described the ability of C. neoformans to undergo hyphal formation and monokaryotic fruiting after an unbudded 2C arrest (54). It is possible that C. neoformans incorporated the cell cycle arrest into a global stress response that ultimately evolved into generation of the titan cell phenotype. Not unsurprisingly, the titan cell phenotype includes traditional aspects of cell cycle regulation, such as ploidy alterations, including ploidy increases during titan cell formation and ploidy reductions during titan cell division, and morphological alterations such as isotropic growth. However, the titan cell phenotype also includes changes linked to defense strategies such as alterations in cell wall, melanin, and capsule structure (19, 22). Cln1 was previously linked to these cell surface phenotypes (51, 52), with the association likely related to the role of Cln1 in the stress cell cycle and its role in cell defense. In support of this conclusion, Garcia-Rodas et al. (51) used the *Galleria* model to bypass the high-temperature growth defect of the *cln1Δ* mutant and showed that the mutant had lower survival inside *Galleria* hemocytes at 30°C, highlighting a critical role for the stress cell cycle and cellular defenses *in vivo*.

Finally, our studies show the unbudded G_2_ arrest and stress cell cycle that gives rise to the polyploid titan cells and subsequent aneuploid daughter cells with novel traits is a preprogrammed response to stress in C. neoformans (20). Given the similarities between the C. neoformans cell cycle and those of multicellular eukaryotes, titan cell formation and aneuploid daughter cell production may be an excellent system to understand the fundamental cellular processes that underlie eukaryotic polyploid cells. The ability to study the cause and consequence of ploidy increases and reductions in a natural, nonengineered, single-celled organism as a model for humans or higher eukaryotes promises immense future research potential.

## MATERIALS AND METHODS

### Ethics statement.

All mice were handled in strict accordance with good animal practice, as defined by the relevant national and/or local animal welfare bodies. All animal work was approved by the University of Minnesota Institutional Animal Care and Use Committee (IACUC) under protocol no. 1308A30852.

### Strains and culture conditions.

The C. neoformans and S. cerevisiae strains used in this study are listed in Table S2 in the supplemental material. KN99α was used as the wild-type strain unless otherwise indicated. Strains were stored in 30% glycerol at −80°C. C. neoformans strains were grown at 30°C in yeast extract-peptone-dextrose (YPD) agar or liquid medium (BD Biosciences, Sparks, MD). S. cerevisiae strains were grown at 30°C in yeast extract-peptone-galactose (YPG) agar or liquid medium. Dropout mix synthetic minus uracil or adenine (US Biological, Salem, MA) with galactose agar was used for S. cerevisiae transformation. YPD agar containing 200 μg/ml nourseothricin (Sigma-Aldrich, St. Louis, MO), 100 μg/ml G-418 (AG Scientific, San Diego, CA), and/or 300 μg/ml hygromycin B (EMD Millipore, Billerica, MA) was used for C. neoformans transformation. Mating was performed on V8 juice agar (67).

10.1128/mBio.02509-21.2 TABLE S2 Strains used in this study. Download Table S2, PDF file, 0.2 MB. Copyright © 2021 Altamirano et al. 2021 Altamirano et al. https://creativecommons.org/licenses/by/4.0/ This content is distributed under the terms of the Creative Commons Attribution 4.0 International license.

### Identification of putative cyclins and cyclin-dependent kinases in C. neoformans.

To identify the putative cyclins in C. neoformans, the protein sequences of S. cerevisiae cyclins (Cln1, Cln2, Cln3, Clb1, Clb2, Clb3, Clb4, Clb5, Clb6, Pcl1, Pcl2, Pcl5, Pcl6, Pcl7, Pcl8, Pcl9, Pcl10, Pho80, Clg1, Ccl1, and Ssn8) and S. cerevisiae CDKs (Cdc28, Pho85, Kin28, Ssn3, and Ctk1) were subjected to BLASTP searches against the C. neoformans var. *grubii* H99 database from the Broad Institute with the E value cutoff set as 1. A second round of BLASTP searches against the C. neoformans var. *grubii* H99 database was carried out using the protein sequences of the putative C. neoformans cyclins and CDKs with E value cutoff set as 1.

### Strain construction.

Primers used in this study are listed in Table S3. The deletion strains were generated by gene disruption as previously described (68). Briefly, the coding region of the putative cyclin or CDK gene was replaced by the nourseothricin *N*-acetyltransferase (*NAT*) drug resistance cassette. An overlap PCR product was created with the 5′ flanking region (∼1 kb), *NAT* resistance cassette, and 3′ flanking region (∼1 kb) (68). The PCR product was introduced into the KN99α strain by biolistic transformation (69). The resulting deletion mutants generated via homologous recombination were confirmed by PCR, sequencing of the PCR product, and Southern blotting. For complementation of the *cln1Δ* deletion strain, an overlap PCR product was generated that contained the KN99α gene promoter, open reading frame (ORF), terminator, and a neomycin (*NEO*) drug resistance cassette (70) and the 3′ flanking region (∼1 kb). The PCR product was introduced into the mutant by biolistic transformation. The resulting complement strain generated via homologous recombination was confirmed by PCR, RT-qPCR, and phenotype analysis.

10.1128/mBio.02509-21.3 TABLE S3 Primers used in this study. Download Table S3, PDF file, 0.2 MB. Copyright © 2021 Altamirano et al. 2021 Altamirano et al. https://creativecommons.org/licenses/by/4.0/ This content is distributed under the terms of the Creative Commons Attribution 4.0 International license.

For generation of the overexpression strains, expression of *CLN1* was driven under the constitutively expressed promoter *GPD1* (33) or copper-repressible promoter *CTR4* (34). An overlap PCR product was generated with 5′ flanking regions (∼1 kb), *NAT* resistance cassette, *GPD1* promoter (0.9 kb), or *CTR4* promoter (2 kb) and 1 kb of the coding region of the KN99α *CLN1* gene (68). The PCR product was introduced into the KN99α strain by biolistic transformation (69). The resulting overexpression strain generated via homologous recombination was confirmed by PCR and RT-qPCR under appropriate expression conditions.

The strain expressing fluorescently labeled *TUB1-GFP* under the endogenous *TUB1* promoter was constructed by first inserting *GFP-TUB1-NEO* with the native TUB1 promoter at the TUB1 locus into the C. neoformans wild-type strain, KN99α. To tag Tub1 with green fluorescent protein (GFP) at the N terminus, the *TUB1* promoter region and *TUB1* gene were first amplified by PCR from C. neoformans KN99α genomic DNA, and the GFP gene was amplified from pAcGFP1 (Clontech, Mountain View, CA). The three PCR fragments were fused together using overlap PCR (68). The overlap PCR product was cloned into pSC-B ampkan (Agilent Technologies, La Jolla, CA) to generate pScGFPTUB1 using a Stratagene blunt PCR cloning kit (Agilent Technologies). The neomycin (*NEO*) drug resistance marker was amplified from pJAF12 and cloned into pSC-B ampkan to make pScNEO. Using the restriction enzyme SpeI, the *NEO* resistance gene was removed from pScNEO and subcloned into pScGFPTUB1 in the SpeI site to create the plasmid pScGFPTUB1-NEO. This plasmid was linearized using the restriction enzyme StuI and biolistically transformed into KN99α (69). The transformants were selected on YPD agar supplemented with 100 μg/ml neomycin. Positive transformants were screened by PCR and fluorescence microscopy.

The *GFP-TUB1-NEO* insertion resulted in two copies of *TUB1* in the KN99α genome. To delete one copy of *TUB1*, disruption cassettes with nourseothricin (*NAT*) drug resistance markers flanked by ∼1-kb upstream and downstream regions of the *TUB1* gene were constructed. The *TUB1* upstream and downstream regions were amplified from the *GFP-TUB1* strain generated above, and the *NAT* drug resistance gene was amplified from plasmid pNATSTM125. Three PCR fragments were then combined using overlap PCR. The overlap PCR product was transformed into the *GFP-TUB1* strain using biolistic transformation. Transformants were selected on YPD agar supplemented with 200 μg/ml nourseothricin. Positive transformants were screened by PCR, and RT-qPCR was performed to confirm that one copy of TUB1 was deleted by comparing the *TUB1* transcript level with the KN99α parental strain (1 copy) and the *GFP-TUB1* strain prior to transformation (2 copies).

Two strains, SL306 (KN99α with *NOP1-mCherry*::*NEO*) and SL321 (KN99**a** with *GFP-NOP1*::*NAT*), were used to generate a KN99**a** strain containing *NOP1-mCherry*::*NEO* by mating (71). Mating was performed on V8 juice agar (pH 7.0) at room temperature in the dark. After 2 weeks, individual basidiospores were micromanipulated onto YPD agar. Strains containing *NOP1-mCherry*::*NEO* were identified using fluorescence microscopy. A mating type **a** version of the *Nop1-mCherry*::*NEO* strain was identified by coculturing with KN99**a** and KN99α tester strains, as described above for mating.

The *NOP1-mCherry*::*NEO GFP-TUB1*::*NAT* strain was generated by mating the KN99a *NOP1-mCherry*::*NEO* with the KN99α *GFP-TUB1*::*NAT* strain. Single spores were isolated by microdissection and cultured on YPD agar containing both NAT and NEO, and the mating type of the resulting progeny was determined as described above. Progeny strains were analyzed by fluorescence microscopy to identify strains with both GFP-labeled microtubules and mCherry-labeled nucleolus.

To generate the *CLN1-His*_6_ strain, an overlap PCR product was created with the *CLN1* ORF with a His tag at the 3′ end (∼1 kb), *GPD1* terminator (∼0.3 kb), *NEO* resistance cassette, and 3′ flanking regions (∼1 kb). The PCR product was introduced into the KN99α strain by biolistic transformation. To generate the *CDK1-Myc* strain, an overlap PCR product was created with the *CDK1* ORF with the Myc tag at the 3′ end (∼1 kb), *GPD1* terminator (∼0.3 kb), *NAT* resistance cassette, and 3′ flanking regions (∼1 kb). The PCR product was introduced into the KN99α strain by biolistic transformation. The resulting deletion mutants generated via homologous recombination were confirmed by PCR and sequencing of the PCR product. To generate the *CLN1-His + CDK1-Myc* strain, a second round of biolistic transformation was carried out by introducing the overlap PCR product of the *CLN1-His*_6_ into the *CDK1-Myc* strain.

### Confirmation of the essential cyclins in C. neoformans.

The diploid strain KN99α/KN99**a** was created as described previously (72) using KN99α *NOP1-mCherry*::*NEO* and KN99**a** *14-3-3-GFP*::*HYG* as the progenitor strains. To generate the KN99**a** *14-3-3-GFP*::*HYG* strain, an overlap PCR product was created with the *14-3-3* ORF (∼1 kb), GFP, *GPD1* terminator (∼0.3 kb), *NEO* resistance cassette, and 3′ flanking regions (∼1 kb). The PCR product was introduced into the KN99**a** strain by biolistic transformation. The resulting strain generated via homologous recombination was confirmed by PCR and sequencing of the PCR product. A coculture of KN99α *NOP1-mCherry*::*NEO* and KN99**a** *14-3-3-GFP*::*HYG* cells was incubated on V8 juice agar overnight at 25°C in the dark. The coculture was transferred to YPD agar containing *NEO* and *HYG* to select the diploid strain KN99α *NOP1-mCherry*::*NEO*/KN99**a** *14-3-3-GFP*::*HYG* at 37°C. The overlap PCR product for the putative essential gene containing the 5′ flanking regions (∼1 kb), *NAT* resistance cassette, and 3′ flanking regions (∼1 kb) was then introduced into the diploid strain by biolistic transformation as described above. The resulting transformants were selected on YPD agar containing NAT, NEO, and HYG at 37°C. The diploid deletion strains were screened by PCR to confirm that only one copy of the candidate gene was disrupted by *NAT*. The occurrence of double-deletion diploid strains was used as evidence that the gene was not essential. Single-deletion diploid strains were then sporulated on V8 juice agar at 25°C in the dark. One hundred basidiospores were microdissected onto YPD agar, and the resulting colonies were screened for *NAT* resistance. Failure to obtain *NAT*-resistant colonies was considered indicative that spores containing the putative essential gene deletion construct were inviable, and these genes are essential in haploid C. neoformans.

### Titan cell formation *in vivo*.

Cells were grown overnight in YPD liquid medium at 30°C, washed three times with phosphate-buffered saline (PBS), and resuspended in PBS at a concentration of 1 × 10^6^ cells/ml based on hemocytometer count. Groups of 6- to 8-week-old female A/J mice (Jackson Laboratory, Bar Harbor, ME) were anesthetized by intraperitoneal pentobarbital injection. Four mice per treatment were infected intranasally with 5 × 10^4^ or 5 × 10^6^ cells in 50 μl PBS. Infected mice were sacrificed at 3 or 14 days postinfection by CO_2_ inhalation. Lungs were removed and then homogenized in 20 ml of Hanks’ balanced salt solution (HBSS) with 0.8 mg/ml collagenase type I (Life Technology, Grand Island, NY). The cell suspension was incubated for 1 h at 37°C with continuous shaking then washed three times with 0.05% SDS to lyse the mammalian cells. The resulting C. neoformans cells were fixed in 3.7% formaldehyde, pelleted, and resuspended in PBS. At least 100 C. neoformans cells per mouse were analyzed for cell body size by microscopy. Cells were classified as typical-sized cells (<10 μm in cell body diameter excluding the capsule) or titan cells (>10 μm in cell body diameter excluding the capsule).

### Cell synchronization.

Cells were grown in 50 ml YPD liquid medium in a 250-ml flask at 30°C, 250 rpm, for 1.5 days to reach stationary phase. The resulting cells were diluted 1:70 with fresh YPD, again as a 50 ml YPD culture in 250 ml flask, at 30°C, 250 rpm, and samples were analyzed at 0 min, 15 min, 30 min, 45 min, 60 min, 75 min, 90 min, 105 min, and 120 min for bud size analysis, flow cytometry, and RT-qPCR.

### Growth curve.

Cells were grown overnight in YPD liquid medium at 30°C and diluted into fresh YPD at a concentration of 5 × 10^4^ cells/ml, 200 μl was pipetted in replicates of 3 into a microplate (tissue culture test plate 96F; TPP, Switzerland), and growth was analyzed using a Synergy H1 hybrid multimode microplate reader (BioTek Instruments, Winooski, VT). Measurements were made every 5 min for 75 min with continuous orbital shaking at 30°C and 37°C. Shaking speed was set to slow and frequency set to 282 (3-mm amplitude). Cell growth was assessed by 600-nm absorbance light scatter measurements. Data were collected using Gen5 Data Analysis Software (BioTek Instruments, Winooski, VT).

### Flow cytometry.

Propidium iodide (PI) staining was used to assess the ploidy of cells. Briefly, cells were washed and suspended in 100 μl of sterile water. Cells were then fixed with 70% ethanol, incubated at 24°C for 1 h, and incubated at 4°C overnight. Cells were then washed and resuspended in 100 μl of RNase A buffer (0.2 M Tris, pH 7.5, 20 mM EDTA) and incubated with 1 μl of RNase A (10 mg/ml) at 37°C for 4 h. After RNase A treatment, cells were washed and resuspended in 900 μl of PBS. A volume of 100 μl PI (0.05 mg/ml) was added to 900 μl of cells and incubated in the dark for 30 min. Immediately prior to analysis, cells were sonicated at 20% amplitude for 5 s. Analysis of cell wall chitin was assessed using calcofluor white staining, as described previously (21). Briefly, cells were fixed in 3.7% formaldehyde, standardized to 1 × 10^6^ cells/ml in PBS, and stained for 5 min at 25°C with 1 μg/ml of calcofluor white (Sigma-Aldrich, St. Louis, MO). Stained cells were pelleted and resuspended in PBS. A total of 10,000 cells for each sample were analyzed using a BD LSR II H4710 or H1160 (Becton, Dickinson, Hercules, CA), and data were analyzed using Flowjo v10 software (TreeStar, Ashland, OR).

Analysis of cell morphology based on the ploidy of the cell was performed using a BD FACSAria II cell sorter. C. neoformans cells were isolated from the lungs of mice 14 days after infection as described above. The cells were then passed over a 20-μm filter to remove the titan cells from the sample, with the flowthrough typically containing a purified population that is >98% typical-sized cells (73). Log-phase cells were grown by inoculating YPD broth medium and incubating the culture overnight at 250 rpm. Stationary-phase cells were grown similarly, but the cultures were incubated for 96 h. All samples were fixed and stained with PI as described above. Cells were gated for singlets and sorted based on PI fluorescence with at least 400,000 cells sorted into each population. A minimum of 100 cells in each of the resulting populations was analyzed for budding morphology using a Zeiss AxioImager microscope (Carl Zeiss, Inc., Thornwood, NY) equipped with ZenBlue2 software (Carl Zeiss, Inc., Thornwood, NY).

### Fluorescence microscopy.

Prior to live cell fluorescence imaging, overnight cultures were pelleted at 15,000 rpm and washed with complete minimal medium (CMM) and resuspended in CMM. For analysis of log-phase and stationary-phase typical-sized cells, cells were spotted and imaged on a 2% agarose patch supplemented with CMM and imaged on a Zeiss AxioImager (Carl Zeiss, Inc., Thornwood, NY). For analysis of *in vivo* titan cells, cells were resuspended in CMM and 500 μl was placed in a 35-mm glass-bottom microwell petri dish (14-mm microwell dish; MatTek Corporation, Ashland, MA). *In vivo* titan cells were then imaged on a Zeiss LSM 800 confocal laser scanning microscope (Carl Zeiss, Inc., Thornwood, NY) or a Zeiss Cell Observer SD spinning disk microscope (Carl Zeiss, Inc., Thornwood, NY). Images were analyzed using ZenBlue2 software (Carl Zeiss, Inc., Thornwood, NY).

### Stationary-phase analysis.

To analyze the ability of cells to enter stationary phase, overnight cultures were diluted to a final concentration of 1 × 10^5^ cells/ml in YPD and incubated at 30°C. At 1, 2, 3, 4, and 5 days after starting the culture, 1 ml of cell suspension was harvested. Cell concentration was enumerated using a hemocytometer, and cell morphology was determined by analysis of at least 100 cells on a Leica DM750 microscope (Leica Biosystems, Wetzlar, Germany). For ploidy analysis, cells were fixed with 70% ethanol, stained with PI, and analyzed by flow cytometry as described above. To assess cells after stationary-phase release, cells were grown for 96 h under nutrient starvation. Cells were then centrifuged to pellet the cells, washed with PBS twice, and resuspended to a final concentration of 2.5 × 10^5^ cells/ml in YPD broth and incubated at 30°C with shaking at 250 rpm. The cell morphology was determined by analysis of at least 100 cells per time point on a Leica DM750 microscope (Leica Biosystems, Wetzlar, Germany).

### Spot assays.

Cultures were washed two times with PBS, resuspended in PBS, and diluted to 2 × 10^6^ cells/ml. The resulting cells were serially diluted 10-fold, and 5 μl of each dilution was spotted onto YPD plates. Plates were incubated at 30°C, and growth was assessed after 48 h of incubation.

### RNA purification and RT-qPCR.

For RT-qPCR analysis of synchronized cell populations, stationary-phase cells were generated after 72 h of incubation as described above. For comparison of *CLN1* RNA levels in titan and typical-sized cells, cell concentration was normalized prior to initiation of the RNA extraction protocol. RNA extraction was performed as previously described (74). Cells were pelleted, frozen in liquid nitrogen, and stored at −80°C. The frozen pellet was lyophilized and vortexed to powder. RNA was extracted using a modified PureZOL RNA isolation reagent/Qiagen procedure. Cells were lysed in 700 μl PureZOL RNA isolation reagent (Bio-Rad, Hercules, CA) and incubated for 5 min at room temperature, and then 140 μl chloroform was added, mixed thoroughly, and centrifuged at 20,000 × *g* for 5 min at room temperature. The aqueous phase was separated and mixed with an equal volume of 70% ethanol and immediately applied to an RNeasy Mini spin column (Qiagen, Valencia, CA). RNA was isolated according to the manufacturer’s protocol. The quality and quantity of RNA were measured using a NanoDrop spectrometer (Thermo Scientific). The iScript one-step RT-PCR kit with SYBR green (Bio-Rad, Hercules, CA) was used for RT-qPCR by following the manufacturer’s protocol. RT-qPCR was performed in an iQ5 real-time PCR detection system (Bio-Rad, Hercules, CA). Gene expression levels were normalized using the endogenous control gene *TEF1* or *ACT1*. Relative copy levels were determined using the comparative threshold cycle (*C_T_*) method (75).

### Co-IP and near-infrared (NIR) Western blot analysis.

The *CLN1-His*_6_ *CDK1-Myc* strain was cultured in 50 ml YPD liquid medium for 1.5 days at 30°C (250 rpm) to reach stationary phase. The culture was diluted 1:70 with fresh YPD and 0-min, 15-min, 30-min, 45-min, 60-min, 75-min, or 90-min time points were collected as described above. Cells were pelleted, frozen in liquid nitrogen, and stored at −80°C. The frozen pellet was lyophilized, vortexed to powder, and suspended in lysis buffer. Cdk1 and its associated proteins were copurified using the Pierce c-Myc-tag immunoprecipitation (IP)/co-IP kit (Thermo Scientific, Rockford, IL). Cell lysates from the *CDK1-Myc* strain were used as a negative control. Cln1-His_6_ and its associated proteins were copurified following the nickel-nitrilotriacetic acid (Ni-NTA) Spin kit procedure (Qiagen, Valencia, CA). Cell lysate from the *CLN1-His*_6_ strain was used as a negative control. The purified proteins were separated by SDS–4 to 15% Mini-PROTEAN TGX precast protein gels (Bio-Rad, Hercules, CA) and then transferred to an Immobilon-FL polyvinylidene difluoride membrane (Merck Millipore Ltd., Tullagreen, Ireland) using a Trans-Blot Turbo transfer system (Bio-Rad, Hercules, CA) for Western blot analysis. The blots were incubated overnight at 4°C with the primary antibodies: His-Tag (D3I1O) XP rabbit monoclonal antibody (MAb) (1/1,000 dilution) (Cell Signaling Technology, Danvers, MA) and Myc-Tag (9B11) mouse MAb (1/1,000 dilution) (Cell Signaling Technology, Danvers, MA), and then incubated for 1 h at room temperature with the secondary antibodies: IRDye 800CW goat anti-rabbit IgG (H+L) (1/20,000 dilution) (LI-COR, Lincoln, NE) and IRDye 680CW goat anti-mouse IgG (H+L) (1/20,000 dilution) (LI-COR, Lincoln, NE). Detection was performed using an Odyssey Fc infrared imaging system (LI-COR, Lincoln, NE).

### Kinase activity assay.

The kinase activity assay was performed as previously described with the following modifications (76). The *CLN1-His*_6_ *CDK1-Myc* strain was cultured in 50 ml YPD liquid medium for 1.5 days at 30°C, 250 rpm, to reach stationary phase. The culture was then diluted 1:70 with fresh YPD and cultured an additional 15 min or 45 min. Cells were pelleted, frozen in liquid nitrogen, stored at −80°C, lyophilized, vortexed to powder, and suspended in lysis buffer, and 2 mg of total protein was incubated with 15 μl goat anti-Myc antibody tag agarose immobilized (Bethyl, Montgomery, TX) at 4°C overnight. The beads were washed three times with cold lysis buffer on a Pierce spin column with screw cap (Thermo Scientific, Rockford, IL) and then suspended in 15 μl 1× kinase buffer. The kinase activity assay was performed with 4 μl beads in a 384-well plate using the ADP-Glo kinase assay (Promega, Madison, WI). The luminescence of each well was read using a Synergy H1 hybrid multimode microplate reader (BioTek Instruments, Winooski, VT). Data were collected using Gen5 Data Analysis Software (BioTek Instruments, Winooski, VT), and the 1× kinase buffer was used as a negative control.

### Phylogenetic and motif analysis of cyclins.

Multiple-sequence alignment of the cyclins was performed using ClustalX2 (77). The neighbor-joining tree was created in MEGA6.06 using the Jones-Taylor-Thornton (JTT) model. Conserved motifs of cyclins were analyzed using MEME Suite 4.10.1 (http://meme-suite.org/index.html). The MEME program was employed using the following parameters: zero or one occurrence per sequence, 10 motifs, motif width set between 6 and 50, site number of each motif set between 2 and 600.

### Complementation of S. cerevisiae cyclin mutants.

An overlap PCR product was created with the S. cerevisiae *GPD1* promoter (865 bp), cDNA of C. neoformans *CLN1* generated by reverse transcription, and the S. cerevisiae *ACT* terminator (319 bp). The PCR product was digested with HindIII and XbaI and then inserted into pRS316 (*URA3* marker) or pRS428 (*ADE1* marker) to generate pRS316-*P_GPD1_*-*CLN1* or pRS428-*P_GPD1_*-*CLN1.* pRS316-*P_GPD1_*-*CLN1* or pRS428-*P_GPD1_*-*CLN1* was introduced into the S. cerevisiae *cln1 cln2 cln3* triple mutant CWY364 (*MAT****a*** *cln1△cln2△cln3△leu*::*GAL-CLN3*::*ade1 his2 trp1-1 ura3 △ns bar1*△), S. cerevisiae *clb3△clb4△clb5△clb6△* quadruple mutant K3418F (*MAT****a*** *ade2-1can1-100 his3-11*,*15 leu2 trp1-1 ura3-1 clb3*::*TRP1 clb4*::*HIS3 clb5*::*hisG clb6*::*LEU2 TRP1*::*GAL-CLB5*), or S. cerevisiae *clb1 clb2 clb3 clb4* quadruple mutant YS108 (*MATα GAL1*::*CLB1* [*LEU2*] *clb1*::*URA3 clb2*::*LEU2 clb3*::*TRP1 clb4*::*HIS2 ade1*) by the LiAc/SS carrier DNA/PEG method (78). Transformants were selected on uracil-free or adenine-free media with galactose and then tested for growth on YPD medium without galactose.

### *In vitro* titan cell formation.

Titan cells were generated *in vitro* as previously described (43). Briefly, cells were grown in 10 ml of YPD for 22 h at 30°C. Cells were then washed with minimal media, resuspended at a final concentration of 1 × 10^6^ cells/ml in minimal media in a 1.5-ml tube, and grown at 30°C with 800 rpm in an Eppendorf Thermomixer (Eppendorf, Hamburg, Germany). Cell size was analyzed after 4 days. For analysis of the affect of *CLN1* expression during *in vitro* titan cell formation, 25 μM copper (Cu^2+^) or 400 μM of the copper chelator bathocuproinedisulfonic acid (BCS) was added during overnight growth and/or during incubation in minimal media.

### Statistical analysis.

Microsoft Excel and GraphPad Prism version 9 were used for statistical analysis. Data sets were analyzed for normality and global tests were performed by analysis of variance (ANOVA) or Kruskal-Wallis with appropriate corrections for multiple comparisons. Pairwise comparisons were carried out by Student's *t* test with Welch’s correction for multiple comparisons. *P* values of ≤0.05 were considered statistically significant.
