# Supplementary material for: The Cyclin Cln1 Controls Polyploid Titan Cell Formation following a Stress-Induced G2 Arrest in Cryptococcus
Source: mBio. 2021 Oct 12;12(5):e02509-21. doi: 10.1128/mBio.02509-21 (PMC8510536; doi:10.1128/mBio.02509-21)
Supplement: TABLE S1 [file mbio.02509-21-st001.pdf]

Supplementary Table S1. Diploid sporulation assay.

Diploid Strain	Number of Spores Microdissected	Number of Colonies Obtained†	Number of Diploid Colonies with Drug Resistance to *NAT* + *NEO*	Number of Haploid Colonies with Drug Resistance (*NAT*/*NEO*)
Wild type (neg control)	100	46	0	0
cln1Δ (pos control)	100	61	1	30
clb2Δ #1	100	18	0	0
ccl1Δ #1	100	12	3	0
sgv1Δ #1	100	13	2	0
kin28Δ #1	50	11	1	0
cdc28Δ #1	150	6	0	0
pho85Δ #1	150	8	7	0

† Incomplete spore germination frequently occurs with *C. neoformans*; thus, the positive and negative control strains were also used to determine relative spore germination frequencies, which were approximately 50% for these experiments.
