# Supplementary material for: The Cyclin Cln1 Controls Polyploid Titan Cell Formation following a Stress-Induced G2 Arrest in Cryptococcus
Source: mBio. 2021 Oct 12;12(5):e02509-21. doi: 10.1128/mBio.02509-21 (PMC8510536; doi:10.1128/mBio.02509-21)
Supplement: FIG S1 [file mbio.02509-21-sf001.pdf]

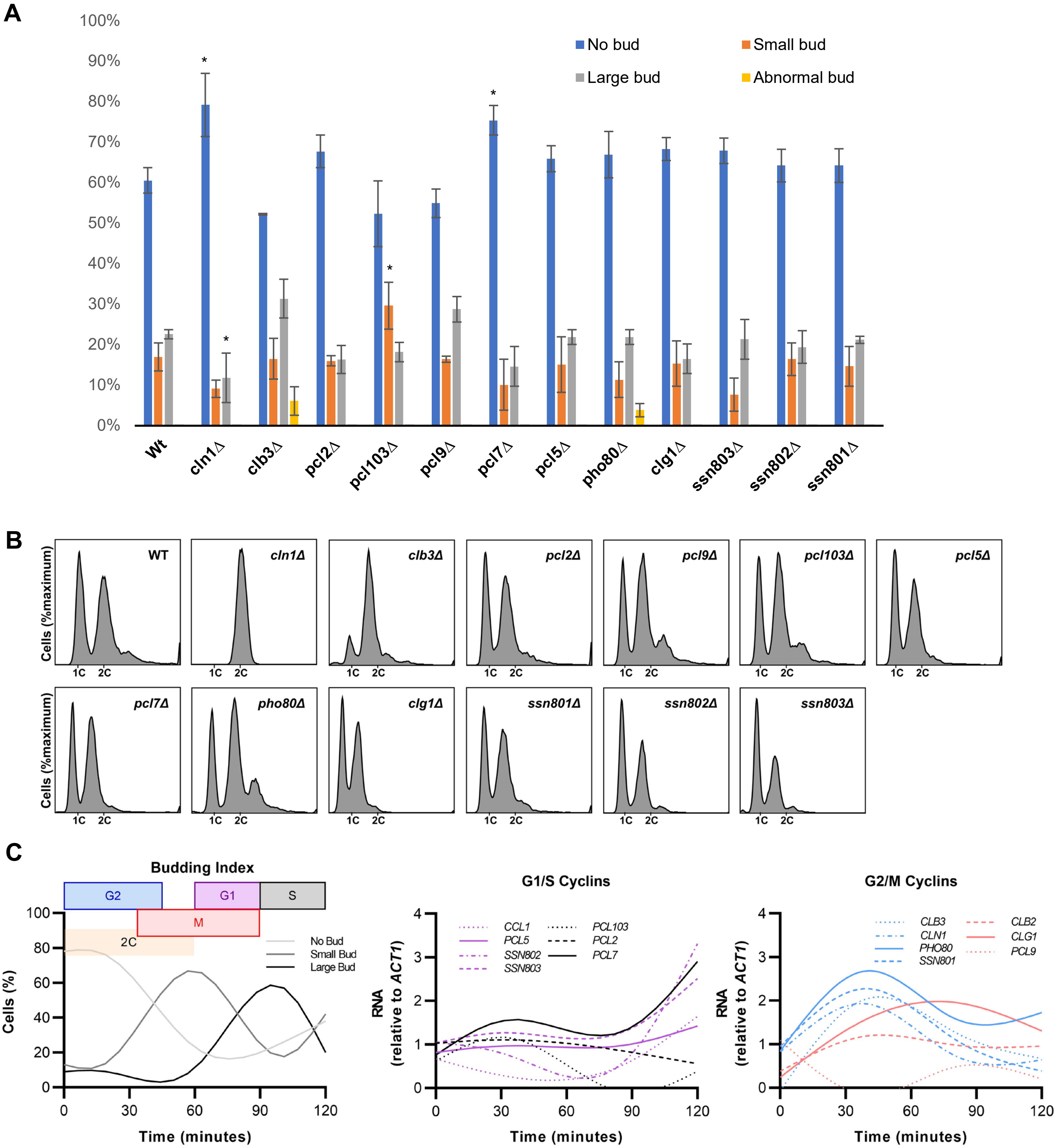

Supplementary Figure SF1. In vitro morphology, ploidy, and expression of cyclin deletion strains. A) Analysis of bud formation in cyclin deletion strains. Cells were grown *in vitro* for 24 hours in YPD media. The morphology of the bud and mother cell was determined for > 300 cells per strain. Cells were classified as having a small bud if the bud size was less than half the size of the mother cell, while the large bud classification refers to a bud size greater than half the size of the mother cell. Error bars indicate SD for three biological replicates. *p<0.05 compared to wild type by two-way ANOVA test with a Dunnett multiplicity correction. **B)** Ploidy analysis of cyclin deletion strains. Cells were grown *in vitro* for 24 hours in YPD media, fixed, and stained with PI for ploidy analysis. **C)** Cell cycle expression analysis of cyclin RNA. RNA levels of wild type cells were analyzed by RT-qPCR to determine the expression of the putative cyclins after release from stationary phase. Budding index and PI staining for ploidy content were assessed at 30-minute intervals after release to estimate the cell cycle stage (colored boxes). After release, cells were 2C for one hour (orange box). Colored boxes designate cell cycle stage based on budding and phenotypic analyses. RNA levels were analyzed at 30-minute intervals and expression levels were normalized to the ACT1 housekeeping gene. Cyclins were grouped based on expression profile into G1/S or G2/M cyclins. Line color corresponds to potential cell cycle stage of each cyclin (purple=G1, black =S, blue=G2, red=M).
