# Supplementary material for: The Cyclin Cln1 Controls Polyploid Titan Cell Formation following a Stress-Induced G2 Arrest in Cryptococcus
Source: mBio. 2021 Oct 12;12(5):e02509-21. doi: 10.1128/mBio.02509-21 (PMC8510536; doi:10.1128/mBio.02509-21)
Supplement: FIG S2 [file mbio.02509-21-sf002.pdf]

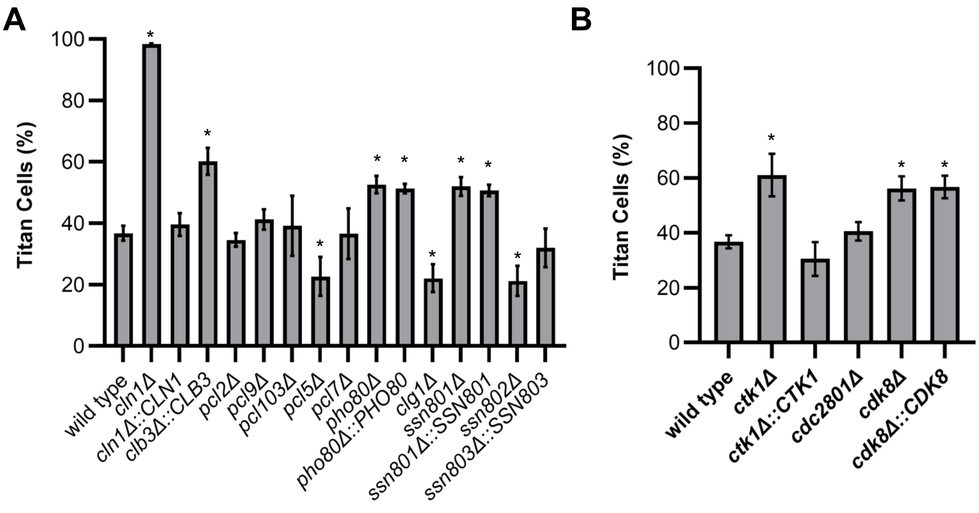

Supplementary Figure SF2. *In vivo* titan formation in cyclin and CDK deletion strains. Mice were infected via inhalation with 5x10^4 cells and titan cell formation in the lungs was analyzed at 3 days post-infection. **A)** *In vivo* titan formation in cyclin deletion strains. The *cln1*Δ mutant exhibits a dramatic increase in titan cell formation that returns to wild type levels upon complementation. The *clb3*Δ and *ssn803*Δ mutants had a severe 37ºC growth defect and there was insufficient recovery from mice for titan cell analysis. However, the *ssn803Δ::SSN803* and *clb3Δ::CLB3* complemented strains were still analyzed for their titan cell phenotypes. The *ssn803Δ::SSN803* complemented strain restored the *in vivo* growth defect and titan cell formation. *The clb3Δ::CLB3* complemented strain only partially restored the *in vivo* growth defect but provided sufficient cells for titan cell analysis and exhibited moderately increased titan cell formation. Both the *pho80*Δ and *ssn801*Δ strains showed increased titan cell formation, but complementation of the gene did not restore wild type levels of titan cell formation, suggesting the titan cell phenotype is not linked to the deletion. **B)** *In vivo* titan formation in CDK deletion strains. The *ctk1*Δ mutant exhibited a moderate increase in titan cell formation that was rescued by complementation. The *cdk8*Δ strain showed increased titan cell formation, but complementation of the gene did not restore wild type levels of titan cell formation suggesting the phenotype was not linked to the *cdk8*Δ deletion. Error bars indicate SD, n ≥ 3 mice per strain. *$p<0.05$ compared to wild type by Student's t-test with Welch's correction for multiple comparisons.
