# Supplementary material for: The Cyclin Cln1 Controls Polyploid Titan Cell Formation following a Stress-Induced G2 Arrest in Cryptococcus
Source: mBio. 2021 Oct 12;12(5):e02509-21. doi: 10.1128/mBio.02509-21 (PMC8510536; doi:10.1128/mBio.02509-21)
Supplement: FIG S3 [file mbio.02509-21-sf003.pdf]

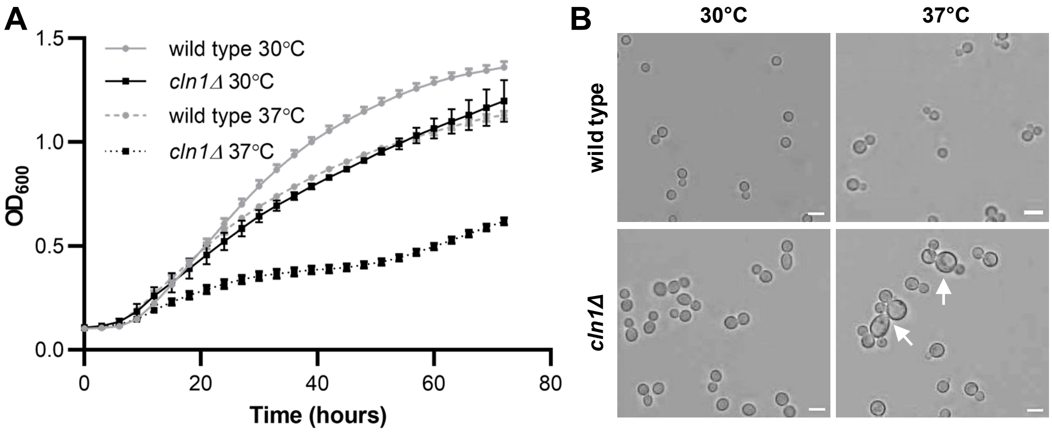

Supplemental Figure SF3. The *cln1Δ* deletion has a growth defect at 37°C. A) All strains were inoculated from a log phase overnight culture into 96-well plates, grown in a shaking spectrophotometer, and analyzed for growth based on optical density at 600 nm every 15 minutes. **B)** Cell morphology was analyzed microscopically after 24 hours growth at 30ºC or 37ºC. White arrows highlight the chains of cells that arose in the *cln1*Δ deletion strain at 37ºC. Scale bar is 10 µm.
