# Supplementary material for: The Cyclin Cln1 Controls Polyploid Titan Cell Formation following a Stress-Induced G2 Arrest in Cryptococcus
Source: mBio. 2021 Oct 12;12(5):e02509-21. doi: 10.1128/mBio.02509-21 (PMC8510536; doi:10.1128/mBio.02509-21)
Supplement: FIG S4 [file mbio.02509-21-sf004.pdf]

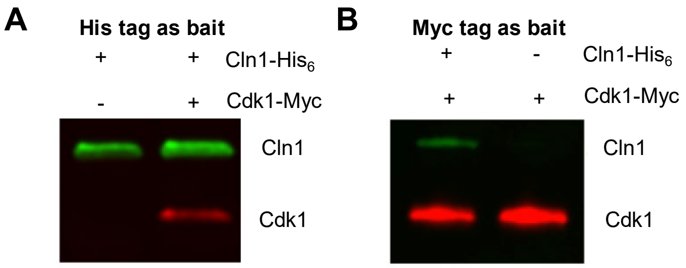

Supplemental Figure SF4. The Cln1 cyclin interacts with the cyclin dependent kinase CDK1. Co-Immunoprecipitation assays were performed using the Cln1-His$_6$ only, Cdk1-Myc only, and the Cln1-His$_6$ Cdk1-Myc double marked strain. **A)** Immunoprecipitation with His to precipitate Cln1-His$_6$, using the Cln1-His6 only strain as the control. The Western blot was then probed with FITC-His and TRITC-Myc labelled antibodies for visualization of Cdk1-Myc co-precipitation with Cln1. **B)** Immunoprecipitation with Myc to precipitate Cdk1-Myc, using the Cdk1-Myc only strain as a control. The Western blot was then probed with FITC-His and TRITC-Myc labelled antibodies for visualization of Cln1-His$_6$ co-precipitation with Cdk1-Myc.
