# Supplementary material for: The Cyclin Cln1 Controls Polyploid Titan Cell Formation following a Stress-Induced G2 Arrest in Cryptococcus
Source: mBio. 2021 Oct 12;12(5):e02509-21. doi: 10.1128/mBio.02509-21 (PMC8510536; doi:10.1128/mBio.02509-21)
Supplement: FIG S5 [file mbio.02509-21-sf005.pdf]

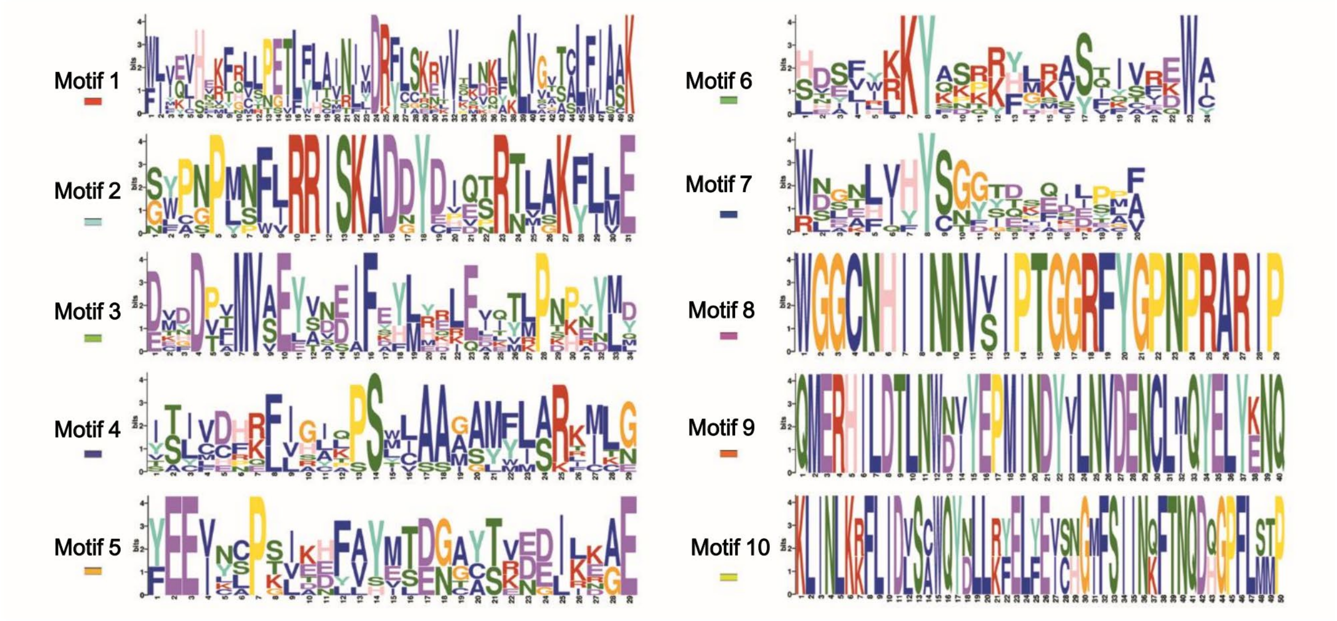

Supplemental Figure SF5. Conserved motif sequences of the Cdc28 cyclins from *C. neoformans* and *S. cerevisiae*. Identification of the 10 protein sequence motifs was performed using MEME Suite 4.10.1 using the following parameters: zero or one occurrence per sequence, 10 motifs, motif width set as between 6 and 50, site number of each motif set as between 2 and 600.
