# Supplementary material for: The Cyclin Cln1 Controls Polyploid Titan Cell Formation following a Stress-Induced G2 Arrest in Cryptococcus
Source: mBio. 2021 Oct 12;12(5):e02509-21. doi: 10.1128/mBio.02509-21 (PMC8510536; doi:10.1128/mBio.02509-21)
Supplement: TABLE S2 [file mbio.02509-21-st002.pdf]

Supplementary Table S2. Strains used in this study.

**Strain Name**	**Genotype**	**Source**
KN99α	MATα wild type reference	(67)
KN99**a**	MAT**a** wild type reference	(67)
cln1Δ	MATα *cln1Δ::NAT*	This study
clb3Δ	MATα *clb3Δ::NAT*	This study
pcl2Δ	MATα *pcl2Δ::NAT*	This study
pcl9Δ	MATα *pcl9Δ::NAT*	This study
pcl103Δ	MATα *pcl103Δ::NAT*	This study
pcl5Δ	MATα *pcl5Δ::NAT*	This study
pcl7Δ	MATα *pcl7Δ::NAT*	This study
pho80Δ	MATα *pho80Δ::NAT*	This study
clg1Δ	MATα *clg1Δ::NAT*	This study
ssn801Δ	MATα *ssn801Δ::NAT*	This study
ssn802Δ	MATα *ssn802Δ::NAT*	This study
ssn803Δ	MATα *ssn803Δ::NAT*	This study
Diploid *cln1Δ*	Diploid, *CLN1/*cln1*Δ::NAT NOP1-mCherry::NEO 14-3-3-GFP::HYG*	This study
Diploid *clb2Δ*	Diploid, *CLB2/*clb2*Δ::NAT NOP1-mCherry::NEO 14-3-3-GFP::HYG*	This study
Diploid *ccl1Δ*	Diploid, *CCL1/ccl1Δ::NAT NOP1-mCherry::NEO 14-3-3-GFP::HYG*	This study
ctk1Δ	MATα *ctk1Δ::NEO*	This study
cdc2801Δ	MATα *cdc28011Δ::NEO*	This study
cdk8Δ	MATα *cdk8Δ::NEO*	This study
Diploid *cdk1Δ*	Diploid*, CDK1/cdkΔ::NEO NOP1-mCherry::NAT 14-3-3-GFP::HYG*	This study
Diploid *sgv1Δ*	Diploid, *SGV1/sgv1Δ::NEO NOP1-mCherry::NAT 14-3-3-GFP::HYG*	This study
Diploid *kin28Δ*	Diploid, *KIN28/kin28Δ::NEO NOP1-mCherry::NAT 14-3-3-GFP::HYG*	This study
Diploid *pho85Δ*	Diploid, *PHO85/pho85Δ::NEO NOP1-mCherry::NAT 14-3-3-GFP::HYG*	This study

Diploid KN99α/KN99**a**	Diploid, MATα *NOP1-mCherry::NEO/*MAT**a** *14-3-3-GFP::HYG*	This study
P_{GPD1}*-CLN1*	MATα P_{GPD1}*-CLN1::NAT*	This study
P_{CTR4}*-CLN1*	MATα, P_{CTR4}*-CLN1::NAT*	This study
cln1Δ::CLN1	MATα *CLN1::NEO*	This study
clb3Δ::CLB3	MATα *CLB3::NEO*	This study
pho80Δ::PHO80	MATα *PHO80::NEO*	This study
ssn801Δ::SSN801	MATα *SSN801::NEO*	This study
ssn803Δ::SSN803	MATα *SSN803::NEO*	This study
ctk1Δ::CTK1	MATα *CTK1::HYG*	This study
cdk8Δ::CDK8	MATα *CDK8::HYG*	This Study
CNV111	MAT**a** *GFP-NDC1::NAT* + *mCherry-CSE4::NEO*	(25)
CWY364	MAT**a** *cln1△cln2△cln3△leu::GAL-CLN3::ade1 his2 trp1-1 ura3 △ns bar1△*	This study; a gift from Dr. Curt Wittenberg
YS108	MATα *GAL1::CLB1 (LEU2) clb1:: URA3 clb2::LEU2 clb3::TRP1 clb4::HIS2 ade1*	(42)
K3418F	MAT**a** *ade2-1can1-100 his3-11,15 leu2 trp1-1 ura3-1 clb3△::TRP1 clb4△::HIS3 clb5△::hisG clb6△::LEU2 TRP1::GAL-CLB5*	This study; a gift from Dr. David T. Stuart
Nop1-mCherry + TUB1-GFP	*NOP1-mCherry::NEO* + *TUB1-GFP::NAT*	This study
CWY364 + P_{GPD1}*-CLN1*	CWY364 + P_{GPD1}*-CLN1::URA3*	This study
YS108 + P_{GPD1}*-CLN1*	YS108 + P_{GPD1}*-CLN1::ADE1*	This study
K3418F + P_{GPD1}*-CLN1*	K3418F + P_{GPD1}*-CLN1::URA3*	This study
CLN1-His$_6$	MATα *CLN1-His*$_6$*::NEO*	This study
CDK1-Myc	MATα *CDK1-Myc::NAT*	This study
CLN1-His + *CDK1-Myc*	MATα *CLN1-His::NEO* + *CDK1-Myc::NAT*	This study
14-3-3-GFP	MAT**a** *14-3-3-GFP::HYG*	This study
