# Supplementary material for: The Cyclin Cln1 Controls Polyploid Titan Cell Formation following a Stress-Induced G2 Arrest in Cryptococcus
Source: mBio. 2021 Oct 12;12(5):e02509-21. doi: 10.1128/mBio.02509-21 (PMC8510536; doi:10.1128/mBio.02509-21)
Supplement: TABLE S3 [file mbio.02509-21-st003.pdf]

Supplementary Table S3. Primers used in this study.

**Strain Name**	**Description**	**Primer Name**	**Sequence**
*clg1*Δ	CNAG_00024 left arm forward	KN0555	GACGCACGCCACATCTCATA
	CNAG_00024 left arm reverse	KN0556	TCTCCAGCTCACATCCTCGGATGTCGGAAGAGGGTGAAA
	NAT for CNAG_00024 forward	KN0557	TTTCACCCTCTTCCGACATCCGAGGATGTGAGCTGGAGA
	NAT for CNAG_00024 reverse	KN0558	GTGGATGGAAGGTGGTTGTGGAAGAGATGTAGAAACTAGCTTCCTGG
	CNAG_00024 right arm forward	KN0559	CCAGGAAGCTAGTTTCTACATCTCTTCCACAACCACCTTCCATCCAC
	CNAG_00024 right arm reverse	KN0560	AGTCATCCTCGTCCTTGCTC
	CNAG_00024+*NAT*+CNAG_00024 Testing forward	KN0561	CGGTCCACTACCAACTCTTA
	CNAG_00024+*NAT*+CNAG_00024 Testing reverse	KN0562	TCATCGCTGTATCGTTCTGG
*pcl2*Δ	CNAG_00183 left arm forward	KN0567	CGTCGTGAAGTTAGTGGAGCC
	CNAG_00183 left arm reverse	KN0568	TCTCCAGCTCACATCCTCGGTTGGTCGTGTATATCAGCCG
	NAT for CNAG_00183 forward	KN0569	CGGCTGATATACACGACCAACCGAGGATGTGAGCTGGAGA
	NAT for CNAG_00183 reverse	KN0570	CGTGAGAAAATGCCAGGGTAGAAGAGATGTAGAAACTAGCTTCCTGG
	CNAG_000183 right arm forward	KN0571	CCAGGAAGCTAGTTTCTACATCTCTTCTACCCTGGCATTTTCTCACG
	CNAG_000183 right arm reverse	KN0572	AACACCGAGCGTCCCGAATG
	CNAG_00183+*NAT*+CNAG_00183 Testing forward	KN0573	CGTCGTGAAGTTAGTGGAGC
	CNAG_00183+*NAT*+CNAG_00183 Testing reverse	KN0574	ACACGCCAGCAGGGTTTCCA
*ssn801*Δ	CNAG_00440 left arm forward	KN0575	CGCATAACACTCACAGAACCG
	CNAG_00440 left arm reverse	KN0576	TCTCCAGCTCACATCCTCGCTAACTGCAGCTTTTATTGGTGTGT
	NAT for CNAG_00440 forward	KN0577	ACACACCAATAAAAGCTGCAGTTAGCGAGGATGTGAGCTGGAGA

	NAT for CNAG_00440 reverse	KN0578	TGACCTATGCTATCTTGCCCGAAGAGATGTAGAAACTAGCTTCCTGG
	CNAG_00440 right arm forward	KN0579	CCAGGAAGCTAGTTTCTACATCTCTTCGGGCAAGATAGCATAGGTCA
	CNAG_00440 right arm reverse	KN0580	CGATGGCTTGAGGATTGGTA
	CNAG_00440+ *NAT*+CNAG_00440 Testing forward	KN0581	TGTCAGAATGAGTGGCGTAG
	CNAG_00440+ *NAT*+CNAG_00440 Testing reverse	KN0582	GCCATCTCGTCTCCCTTGTC
pho80Δ	CNAG_01922 left arm forward	KN0583	CTGGAATACTGTAGCATCACCTGTC
	CNAG_01922 left arm reverse	KN0584	TCTCCAGCTCACATCCTCGGGTGGCTATGGCGTGCTTG
	NAT for CNAG_01922 forward	KN0585	CAAGCACGCCATAGCCACCCGAGGATGTGAGCTGGAGA
	NAT for CNAG_01922 reverse	KN0586	GAATGGAGATACGGCAGGATGAAGAGATGTAGAAACTAGCTTCCTGG
	CNAG_01922 right arm forward	KN0587	CCAGGAAGCTAGTTTCTACATCTCTTCATCCTGCCGTATCTCCATTC
	CNAG_01922 right arm reverse	KN0588	AGCGCTCGCCTTGTGAACTA
	CNAG_01922+ *NAT*+CNAG_01922 Testing forward	KN0589	CGAAGGGGAATGCTGGACG
	CNAG_01922+ *NAT*+CNAG_01922 Testing reverse	KN0590	CACACCGCCCCAATAGACAAG
clb3Δ	CNAG_02095 left arm forward	KN0591	AATCCGTGAAGGGAATGTGG
	CNAG_02095 left arm reverse	KN0592	TCTCCAGCTCACATCCTCGGGGTCGCAGATACTTACTCG
	NAT for CNAG_02095 forward	KN0593	CGAGTAAGTATCTGCGACCCCGAGGATGTGAGCTGGAGA
	NAT for CNAG_02095 reverse	KN0594	TACAGCCCAAGGTTAGCCCAGAAGAGATGTAGAAACTAGCTTCCTGG
	CNAG_02095 right arm forward	KN0595	CCAGGAAGCTAGTTTCTACATCTCTTCTGGGCTAACCTTGGGCTGTA
	CNAG_02095 right arm reverse	KN0596	TTACCTGCGATGACCTTTGG
	CNAG_02095+ *NAT*+CNAG_02095 Testing forward	KN0597	CGAATGAGATACAGGGTTGC

	CNAG_02095+ *NAT*+CNAG_02095 Testing reverse	KN0598	CAGTTCCTCCAATACCTCCT
ssn802Δ	CNAG_02127 left arm forward	KN0599	TAAAGAGGATTGGACTCGTGC
	CNAG_02127 left arm reverse	KN0600	TCTCCAGCTCACATCCTCGGCTGCTGTATATCCAGGCCCT
	NAT for CNAG_02127 forward	KN0601	AGGGCCTGGATATACAGCAGCCGAGGATGTGAGCTGGAGA
	NAT for CNAG_02127 reverse	KN0602	ACATAGAAGACACAGAAATACCAACGAAGAGATGTAGAAACTAGCTTCCTGG
	CNAG_02127 right arm forward	KN0603	CCAGGAAGCTAGTTTCTACATCTCTTCGTTGGTATTTCTGTGTCTTCTATGT
	CNAG_02127 right arm reverse	KN0604	GCCCGACGCAGGTGTTTGT
	CNAG_02127+ *NAT*+CNAG_02127 Testing forward	KN0605	TCTCCTCCTGTTGCTTGGTG
	CNAG_02127+ *NAT*+CNAG_02127 Testing reverse	KN0606	CTCCTCCCGAAGGTTACTCC
pcl5Δ	CNAG_02658 left arm forward	KN0607	CCAAACGACCGACTCCTG
	CNAG_02658 left arm reverse	KN0608	TCTCCAGCTCACATCCTCGCCCCGTCTAATCAAACCC
	NAT for CNAG_02658 forward	KN0609	GGGTTTGATTAGACGGGGCGAGGATGTGAGCTGGAGA
	NAT for CNAG_02658 reverse	KN0610	ACGATGTTGGAGGATGGAGGGAAGAGATGTAGAAACTAGCTTCCTGG
	CNAG_02658 right arm forward	KN0611	CCAGGAAGCTAGTTTCTACATCTCTTCCCTCCATCCTCCAACATCGT
	CNAG_02658 right arm reverse	KN0612	CTTCACCTTACCTATCCTCC
	CNAG_02658+ *NAT*+CNAG_02658 Testing forward	KN0613	CGAGCCGATAATACTACGCC
	CNAG_02658+ *NAT*+CNAG_02658 Testing reverse	KN0614	TATGCCCAGGACACCGTAGA
pcl103Δ	CNAG_03385 left arm forward	KN0615	TCTGGCAGCGTGGGTTCATA
	CNAG_03385 left arm reverse	KN0616	TCTCCAGCTCACATCCTCGTCGGGAGAACGACAGAATGGTA
	NAT for CNAG_03385 forward	KN0617	TACCATTCTGTCGTTCTCCCGACGAGGATGTGAGCTGGAGA

	NAT for CNAG_03385 reverse	KN0618	CGGTAGGAGCGACTTTGGATGAAGAGATGTAGAAACTAGCTTCCTGG
	CNAG_03385 right arm forward	KN0619	CCAGGAAGCTAGTTTCTACATCTCTTCATCCAAAGTCGCTCCTACCG
	CNAG_03385 right arm reverse	KN0620	TACTGAGCCTCCGACCAAAT
	CNAG_03385+NAT+CNAG_03385 Testing forward	KN0621	GAACGGTCGGTTACGGTGGA
	CNAG_03385+NAT+CNAG_03385 Testing reverse	KN0622	TAGCAGACCATCGGCTTTGA
Diploid *ccl1*Δ	CNAG_04405 left arm forward	KN0623	CTGGGCTACAATGGACAAGA
	CNAG_04405 left arm reverse	KN0624	TCTCCAGCTCACATCCTCGTGAGTGCTGAGACGGAGAAA
	NAT for CNAG_04405 forward	KN0625	TTTCTCCGTCTCAGCACTCACGAGGATGTGAGCTGGAGA
	NAT for CNAG_04405 reverse	KN0626	CTTTAGCCGCTGCGTCTGGAAGAGATGTAGAAACTAGCTTCCTGG
	CNAG_04405 right arm forward	KN0627	CCAGGAAGCTAGTTTCTACATCTCTTCCAGACGCAGCGGCTAAAG
	CNAG_04405 right arm reverse	KN0628	AAGCCCACCATCGTCTGA
	CNAG_04405+*NAT*+CNAG_04405 Testig forward	KN0629	GTCGTGCCTCGGGATAATGA
	CNAG_04405+*NAT*+CNAG_04405 Testig reverse	KN0630	ACGCACCTATTCGTGACAGA
Diploid *clb2*Δ	CNAG_04575 left arm forward	KN0631	GGGTAGAAAGTTGTCAGGTGGG
	CNAG_04575 left arm reverse	KN0632	TCTCCAGCTCACATCCTCGTAAATGTCAGCTTGAGTTTAGATGT
	NAT for CNAG_04575 forward	KN0633	ACATCTAAACTCAAGCTGACATTTACGAGGATGTGAGCTGGAGA
	NAT for CNAG_04575 reverse	KN0634	CAAGCGCTCCTTGTCGCATGAAGAGATGTAGAAACTAGCTTCCTGG
	CNAG_04575 right arm forward	KN0635	CCAGGAAGCTAGTTTCTACATCTCTTCATGCGACAAGGAGCGCTTG
	CNAG_04575 right arm reverse	KN0636	CTCTACCCATTTATCCTGGTCTCA
	CNAG_04575+*NAT*+CNAG_04575 Teting forward	KN0637	CAGCACGCAGTCTCGTAAGT
	CNAG_04575+*NAT*+CNAG_04575 Teting reverse	KN0638	GGGTGGGAAGAAGGTTTGAG
*pcl7*Δ	CNAG_05524 left arm forward	KN0639	TGTGGTAGTTTCGGACATAGTTCAT

	CNAG_05524 left arm reverse	KN0640	TCTCCAGCTCACATCCTCGCGAATGGTATATCAAGGTCTGTGG
	NAT for CNAG_05524 forward	KN0641	CCACAGACCTTGATATACCATTCGCGAGGATGTGAGCTGGAGA
	NAT for CNAG_05524 reverse	KN0642	AACCCAGCAAGCACTTTCAGGAAGAGATGTAGAAACTAGCTTCCTGG
	CNAG_05524 right arm forward	KN0643	CCAGGAAGCTAGTTTCTACATCTCTTCCTGAAAGTGCTTGCTGGGTT
	CNAG_05524 right arm reverse	KN0644	GAAGGGCTCAAGGCATACTA
	CNAG_05524+*NAT*+CNAG_05524 Testing forward	KN0645	GCAGCATTCGCTCTGTTGGT
	CNAG_05524+*NAT*+CNAG_05524 Testing reverse	KN0646	ATCCTCCTGCGTGATGCCTA
ssn803Δ	CNAG_05901 left arm forward	KN0647	GGAGGTAACCCTTGATGTGG
	CNAG_05901 left arm reverse	KN0648	TCTCCAGCTCACATCCTCGGTAGAAGCCCTATGGGAGAT
	NAT for CNAG_05901 forward	KN0649	ATCTCCCATAGGGCTTCTACCGAGGATGTGAGCTGGAGA
	NAT for CNAG_05901 reverse	KN0650	GGGTGTCAGAAGTCTCAGCAGAAGAGATGTAGAAACTAGCTTCCTGG
	CNAG_05901 right arm forward	KN0651	CCAGGAAGCTAGTTTCTACATCTCTTCTGCTGAGACTTCTGACACCC
	CNAG_05901 right arm reverse	KN0652	ACTCGTCCAACAATCGCTCA
	CNAG_05901+*NAT*+CNAG_05901 forward	KN0653	GCCTCACCAACAGTCTTCTT
	CNAG_05901+NAT+CNAG_05901 reverse	KN0654	GTGACCACGAAGAAAACAGG
cln1Δ	CNAG_06092 left arm forward	KN0655	GCTTTACGATTAGTTCTGGCTTCTG
	CNAG_06092 left arm reverse	KN0656	TCTCCAGCTCACATCCTCGTTTGGGTGGACTTTCGCAGG
	NAT for CNAG_06092 forward	KN0657	CCTGCGAAAGTCCACCCAAACGAGGATGTGAGCTGGAGA
	NAT for CNAG_06092 reverse	KN0658	GGAATAACCTTAGCCATCGGGAAGAGATGTAGAAACTAGCTTCCTGG
	CNAG_06092 right arm forward	KN0659	CCAGGAAGCTAGTTTCTACATCTCTTCCCGATGGCTAAGGTTATTCC
	CNAG_06092 right arm reverse	KN0660	CGGGCGAAGAACGAAGAAGA
	CNAG_06092+NAT+CNAG_06092 Testing forward	KN0661	GGACGGGATGGAGAATGAGG

	CNAG_06092+NAT+CNAG_06092 Testing reverse	KN0662	GTTATGGGTTGAGAAGGGTG
pcl9Δ	CNAG_00442 left arm forward	KN0663	GTGGGACTCTTTCACTTGCTCT
	CNAG_00442 left arm reverse	KN0664	TCTCCAGCTCACATCCTCGAGTTGAGTCGCAATGATTGTCGGTA
	NAT for CNAG_00442 forward	KN0665	TACCGACAATCATTGCGACTCAACTCGAGGATGTGAGCTGGAGA
	NAT for CNAG_00442 reverse	KN0666	GTCCTCCCAGCCCTCTTGAAGAAGAGATGTAGAAACTAGCTTCCTGG
	CNAG_00442 right arm forward	KN0667	CCAGGAAGCTAGTTTCTACATCTCTTCTTCAAGAGGGCTGGGAGGAC
	CNAG_00442 right arm reverse	KN0668	GTCCATACGAGGTGTCAGCA
	CNAG_00442+NAT+CNAG_00442 Testing forward	KN0669	AGCCCGAGTCAGAAGTCAAC
	CNAG_00442+NAT+CNAG_00442 Testing reverse	KN0670	GTATCTTTCCGTGACCTGCT
cdc2801Δ	CNAG_00415 left arm forward	KN0671	GCAGTATTCAGCCCTAACCT
	CNAG_00415 left arm reverse	KN0672	TCTCCAGCTCACATCCTCGCTGGGTGTGTTTGACTGAAATATG
	NEO for CNAG_00415 forward	KN0673	CATATTTCAGTCAAACACACCCAGCGAGGATGTGAGCTGGAGA
	NEO for CNAG_00415 reverse	KN0674	AGAACGCACGAGAAGTATGGGGTTTATCTGTATTAACACGG
	CNAG_00415 right arm forward	KN0675	CCGTGTTAATACAGATAAACCCCATACTTCTCGTGCGTTCT
	CNAG_00415 right arm reverse	KN0676	GTTCCTCTGACGCTTCTATC
	CNAG_00415+NEO+CNAG_00415 Testing forward	KN0677	GCCTCCGTCACCATTCGCTAT
	CNAG_00415+NEO+CNAG_00415 Testing reverse	KN0678	TACATCGGGGTGCTGTCAAT
Diploid *sgv1Δ*	CNAG_05549 left arm forward	KN0733	TCGGAGCACTGAGGAAAGCC
	CNAG_05549 left arm reverse	KN0734	TCTCCAGCTCACATCCTCGAACTGGGTAAGTCCGCAATGGT
	NEO for CNAG_05549 forward	KN0735	ACCATTGCGGACTTACCCAGTTCGAGGATGTGAGCTGGAGA
	NEO for CNAG_05549 reverse	KN0736	CCTACTCGGCGATTCCATCAATGGTTTATCTGTATTAACACGG
	CNAG_05549 right arm forward	KN0737	CCGTGTTAATACAGATAAACCATTGATGGAATCGCCGAGTAGG

	CNAG_05549 right arm reverse	KN0738	TGAAACGAGGGAGTGATAAGGATT
	CNAG_05549+NEO+CNAG_05549 Testing forward	KN0739	TCGCCCATACCTTCTTACTCTC
	CNAG_05549+NEO+CNAG_05549 Testing reverse	KN0740	GGAGACGGAGTCGGTTTCAG
Diploid *cdk1*Δ	CNAG_01664 left arm forward	KN0687	GCGTCGTAGTGGGTGTAGATG
	CNAG_01664 left arm reverse	KN0688	TCTCCAGCTCACATCCTCGGGTGTTTTTCGATCGTGGG
	NEO for CNAG_01664 forward	KN0689	CCCACGATCGAAAAACACCCGAGGATGTGAGCTGGAGA
	NEO for CNAG_01664 reverse	KN0690	GTAGGCTTGTAATCGGGCAAGGTTTATCTGTATTAACACGG
	CNAG_01664 right arm forward	KN0691	CCGTGTTAATACAGATAAACCTTGCCCGATTACAAGCCTAC
	CNAG_01664 right arm reverse	KN0692	TGGGGAAGGACAGTTTGAAGAG
	CNAG_01664+NEO+CNAG_01664 Testng forward	KN0693	GCGTCGTAGTGGGTGTAGAT
	CNAG_01664+NEO+CNAG_01664 Testng reverse	KN0694	CCACCCTCCGAAGAAACCGT
*ctk1*Δ	CNAG_04118 left arm forward	KN0695	GCATGGTCAGATGAGGGCTAA
	CNAG_04118 left arm reverse	KN0696	TCTCCAGCTCACATCCTCGGGCGGATGTATTCCAATTTTT
	NEO for CNAG_04118 forward	KN0697	AAAAATTGGAATACATCCGCCCGAGGATGTGAGCTGGAGA
	NEO for CNAG_04118 reverse	KN0698	TTTTACCCTTCATTCTCCTCCTTCGGTTTATCTGTATTAACACGG
	CNAG_04118 right arm forward	KN0699	CCGTGTTAATACAGATAAACCGAAGGAGGAGAATGAAGGGTAAAA
	CNAG_04118 right arm reverse	KN0700	CAGCGTCTGTCCGTCATCACTC
	CNAG_04118+*NEO*+CNAG_04118 Testing forward	KN0701	AGTGACATCCAGGACAATCG
	CNAG_04118+*NEO*+CNAG_04118 Testing reverse	KN0702	AGGGAGGAATGTGATGTGGT
*ssn3*Δ	CNAG_06086 left arm forward	KN0703	GCACTTCCATCGTGGCTATAC
	CNAG_06086 left arm reverse	KN0704	TCTCCAGCTCACATCCTCGTTTGTTGTATTCCAGCCTTATTGCC
	NEO for CNAG_06086 forward	KN0705	GGCAATAAGGCTGGAATACAACAAACGAGGATGTGAGCTGGAGA

	NEO for CNAG_06086 reverse	KN0706	CCCATCTTAGCATCACCGTTGGTTTATCTGTATTAACACGG
	CNAG_06086 right arm forward	KN0707	CCGTGTTAATACAGATAAACCAACGGTGATGCTAAGATGGG
	CNAG_06086 right arm reverse	KN0708	CCTTACCGACCGTTCCTTCA
	CNAG_06086+*NEO*+CNAG_06086 Testing forward	KN0709	CATTGTCGGACTCGTCTTCA
	CNAG_06086+NEO+CNAG_06086 Testing reverse	KN0710	ACGATACCACCACTCCTTTG
Diploid *kin28*Δ	CNAG_06445 left arm forward	KN0711	ACCCGAGCGGATTCAAGAG
	CNAG_06445 left arm reverse	KN0712	TCTCCAGCTCACATCCTCGTGTTATACAATGTCGCGGGTATCTA
	NEO for CNAG_06445 forward	KN0713	TAGATACCCGCGACATTGTATAACACGAGGATGTGAGCTGGAGA
	NEO for CNAG_06445 reverse	KN0714	GCTTCCCTTGAGTTTCATCCGGTTTATCTGTATTAACACGG
	CNAG_064456 right arm forward	KN0715	CCGTGTTAATACAGATAAACCGGATGAAACTCAAGGGAAGC
	CNAG_064456 right arm reverse	KN0716	AACCATCGGTCTACCACTCC
	CNAG_06445+NEO+CNAG_06445 Testing forward	KN0717	TCACCCGAGCGGATTCAAGA
	CNAG_06445+NEO+CNAG_06445 Testing reverse	KN0718	TGCGATAGTCATAGGGTAGG
Diploid *pho85*Δ	CNAG_08022 left arm forward	KN0719	GATTATCGGGCGAGTCAGGC
	CNAG_08022 left arm reverse	KN0720	TCTCCAGCTCACATCCTCGGCCGAACGAATTAGCTCCTG
	NEO for CNAG_08022 forward	KN0721	CAGGAGCTAATTCGTTCGGCCGAGGATGTGAGCTGGAGA
	NEO for CNAG_08022 reverse	KN0722	GCTGTCCCATCATCATCTCAGGTTTATCTGTATTAACACGG
	CNAG_08022 right arm forward	KN0723	CCGTGTTAATACAGATAAACCTGAGATGATGATGGGACAGC
	CNAG_08022 right arm reverse	KN0724	TTGCCTACGAACGACAGAAG
	CNAG_08022+*NEO*+CNAG_08022 Testing forward	KN0725	TTATCGGGCGAGTCAGGCAG
	CNAG_08022+*NEO*+CNAG_08022 Testing reverse	KN0726	TTGCTGACCTGCGTTTGCTT
*cln1*Δ*::CLN1*	CNAG_06092 ORF forward	KN0655	GCTTTACGATTAGTTCTGGCTTCTG

	CNAG_06092 ORF reverse	KN1003	TCTCCAGCTCACATCCTCGCTGGGAATGAAGGGTACGTACAAAA
	Neo for CNAG_06092 forward	KN1031	TTTTGTACGTACCCTTCATTCCCAGCGAGGATGTGAGCTGGAGA
	Neo for CNAG_06092 reverse	KN0658	GGAATAACCTTAGCCATCGGGAAGAGATGTAGAAACTAGCTTCCTGG
	CNAG_06092 right arm forward	KN0659	CCAGGAAGCTAGTTTCTACATCTCTTCCCGATGGCTAAGGTTATTCC
	CNAG_06092 right arm reverse	KN0660	CGGGCGAAGAACGAAGAAGA
*clb3*Δ:: *CLB3*	CNAG_02095 ORF forward	KN0591	AATCCGTGAAGGGAATGTGG
	CNAG_02095 ORF reverse	KN1005	TCTCCAGCTCACATCCTCGCACCGCTTTTTATTGGTACTATGG
	Neo for CNAG_02095 forward	KN1036	CCATAGTACCAATAAAAAGCGGTGCGAGGATGTGAGCTGGAGA
	Neo for CNAG_02095 reverse	KN0594	TACAGCCCAAGGTTAGCCCAGAAGAGATGTAGAAACTAGCTTCCTGG
	CNAG_02095 right arm forward	KN0595	CCAGGAAGCTAGTTTCTACATCTCTTCTGGGCTAACCTTGGGCTGTA
	CNAG_02095 right arm reverse	KN0596	TTACCTGCGATGACCTTTGG
*pho80*Δ:: *PHO80*	CNAG_01922 ORF forward	KN0583	CTGGAATACTGTAGCATCACCTGTC
	CNAG_01922 ORF reverse	KN1004	TCTCCAGCTCACATCCTCGTCTATTCGTCCTACTACCTTTGAGC
	Neo for CNAG_01922 forward	KN1033	GCTCAAAGGTAGTAGGACGAATAGACGAGGATGTGAGCTGGAGA
	Neo for CNAG_01922 reverse	KN0586	GAATGGAGATACGGCAGGATGAAGAGATGTAGAAACTAGCTTCCTGG
	CNAG_01922 right arm forward	KN0587	CCAGGAAGCTAGTTTCTACATCTCTTCATCCTGCCGTATCTCCATTC
	CNAG_01922 right arm reverse	KN0588	AGCGCTCGCCTTGTGAACTA
*ssn801*Δ:: *SSN801*	CNAG_00440 ORF forward	KN0575	GTAATGTATCCCTGATGCCG
	CNAG_00440 ORF reverse	KN0794	TCTCCAGCTCACATCCTCGCAACAAGTTCGTGCCCTACC
	NEO for CNAG_00440 forward	KN0795	GGTAGGGCACGAACTTGTTGCGAGGATGTGAGCTGGAGA
	NEO for CNAG_00440 reverse	KN0578	TGACCTATGCTATCTTGCCCGAAGAGATGTAGAAACTAGCTTCCTGG
	CNAG_00440 right arm forward	KN0579	CCAGGAAGCTAGTTTCTACATCTCTTCGGGCAAGATAGCATAGGTCA
	CNAG_00440 right arm reverse	KN0580	CGATGGCTTGAGGATTGGTA

ssn803∆::SSN803	CNAG_05901 ORF forward	KN0647	GGAGGTAACCCTTGATGTGG
	CNAG_05901 ORF reverse	KN0818	TCTCCAGCTCACATCCTCGCAACCCTTACACCCTGACTGA
	NEO for CNAG_05901 forward	KN0819	TCAGTCAGGGTGTAAGGGTTGCGAGGATGTGAGCTGGAGA
	NEO for CNAG_05901 reverse	KN0650	GGGTGTCAGAAGTCTCAGCAGAAGAGATGTAGAAACTAGCTTCCTGG
	CNAG_05901 right arm forward	KN0651	CCAGGAAGCTAGTTTCTACATCTCTTCTGCTGAGACTTCTGACACCC
	CNAG_05901 right arm reverse	KN0652	ACTCGTCCAACAATCGCTCA
ctk1∆::CTK1	CNAG_04118 ORF forward	KN0695	CAGATGATGGAGCGTGTTTC
	CNAG_04118 ORF reverse	KN0826	TCTCCAGCTCACATCCTCGGAAGGGAGCCATCCTAACCAC
	HYG for CNAG_04118 forward	KN0827	GTGGTTAGGATGGCTCCCTTCCGAGGATGTGAGCTGGAGA
	HYG for CNAG_04118 reverse	KN0698	AGTTCTTTGACCGATGGCGGTTTATCTGTATTAACACGG
	CNAG_04118 right arm forward	KN0699	CCGTGTTAATACAGATAAACCGCCATCGGTCAAAGAACT
	CNAG_04118 right arm reverse	KN0700	AGGGAGCCATCCTAACCA
ssn3∆::SSN3	CNAG_06086 ORF forward	KN1055	TGCACTTCCATCGTGGCTATAC
	CNAG_06086 ORF reverse	KN1056	TCTCCAGCTCACATCCTCGGAAGTTATCCTGTAATCCTCGCG
	H*YG* for CNAG_06086 forward	KN1057	CGCGAGGATTACAGGATAACTTCCGAGGATGTGAGCTGGAGA
	HYG for CNAG_06086 reverse	KN1058	CCCATCTTAGCATCACCGTTAACTAGCTTCCTGGTTTCAGAGACA
	CNAG_06086 right arm forward	KN1059	TGTCTCTGAAACCAGGAAGCTAGTTAACGGTGATGCTAAGATGGG
	CNAG_06086 right arm reverse	KN0710	ACGATACCACCACTCCTTTG
14-3-3-GFP	14-3-3 ORF forward	KN0775	GCAGGCTGAGCGATACGAAG
	14-3-3 ORF reverse	KN0776	CTCCTCGCCCTTGCTCACAGCCGCAGGGGCAACCTC
	GFP Forward	KN0777	GAGGTTGCCCCTGCGGCTGTGAGCAAGGGCGAGGAG
	GFP Reverse	KN0752	TCTGCTACTGTAACCCCCTCTAGTACAGCTCGTCCATGCCGT
	GPD1 terminator Forward	KN0753	ACGGCATGGACGAGCTGTACTAGAGGGGGTTACAGTAGCAGA
	GPD1 terminator Reverse	KN0754	TCTCCAGCTCACATCCTCGGCACAAGGGTCTCAGGGATG

	NEO forward	KN0755	CATCCCTGAGACCCTTGTGCCGAGGATGTGAGCTGGAGA
	NEO reverse	KN0778	GCCCCATAACACCCGAACGATAAACTAGCTTCCTGGTTTCAGAGACA
	14-3-3 right arm forward	KN0779	TGTCTCTGAAACCAGGAAGCTAGTTTATCGTTCGGGTGTTATGGGGC
	14-3-3 right arm reverse	KN0780	GCGAGCCTGAAGGAGTATTTGG
PGPD1-CLN1	CNAG_06092 promoter + NAT forward	KN0655	GCTTTACGATTAGTTCTGGCTTCTG
	CNAG_06092 promoter + NAT reverse	KN1050	AGATATCAGAAAGTCGCTGGAACATAACTAGCTTCCTGGTTTCAGAGACA
	GPD1 promoter forward	KN1051	TGTCTCTGAAACCAGGAAGCTAGTTATGTTCCAGCGACTTTCTGATATCT
	GPD1 promoter reverse	KN1052	CTGTAGGCGCAACGGGCATTGTATTTATGCAAGTATACTCCTAG
	CNAG_06092 ORF forward	KN1053	CTAGGAGTATACTTGCATAAATACAATGCCCGTTGCGCCTACAG
	CNAG_06092 ORF reverse	KN1041	AATGACGATTTCTGACACTTTCTC
PCTR4-CLN1	CNAG_06092 promoter + *NAT* forward	KN0655	GCTTTACGATTAGTTCTGGCTTCTG
	CNAG_06092 promoter + *NAT* reverse	KN1037	GACGTTCATTTACCAGCAAAAATACAACTAGCTTCCTGGTTTCAGAGACA
	Ctr4 promoter forward	KN1038	TGTCTCTGAAACCAGGAAGCTAGTTGTATTTTTGCTGGTAAATGAACGTC
	Ctr4 promoter reverse	KN1039	CTGTAGGCGCAACGGGCATGATGGATGGTGTCCTTTCGTAA
	CNAG_06092 ORF forward	KN1040	TTACGAAAGGACACCATCCATCATGCCCGTTGCGCCTACAG
	CNAG_06092 ORF reverse	KN1041	AATGACGATTTCTGACACTTTCTC
CWY364 (*PGPD1-CLN1*), YS108 (*PGPD1-CLN1*), K3418F (*PGPD1-CLN1*)	*Saccharomyces cerevisiae GPD1* promoter forward	KN1112	GCGATAAGCTTAAAAGAAAGCCAAGCGTGTAGAC
	Saccharomyces cerevisiae GPD1 promoter reverse	KN1061	CTGTAGGCGCAACGGGCATCTTTATATTATCAATATTTGTGTTTGTGG
	CNAG_06092 ORF forward	KN1062	CCACAAACACAAATATTGATAATATAAAGATGCCCGTTGCGCCTACAG
	CNAG_06092 ORF reverse	KN1063	TACGCGCACAAAAGCAGAGACTAATTGCTTAGCCGTCTCATAAC
	Saccharomyces cerevisiae terminator forward	KN1064	GTTATGAGACGGCTAAGCAATTAGTCTCTGCTTTTGTGCGCGTA
	Saccharomyces cerevisiae terminator reverse	KN1113	CACGTTCTAGAAACTGAAAAGCGATGAAGAGATGAT
CDK1-Myc	CNAG_01664 ORF forward	KN1078	TGCCAAGCACTTCGATCAGAG

	CNAG_01664 ORF-Myc reverse	KN1171	CAGATCCTCTTCTGAGATGAGTTTTTGTTCTGCGGCGGCGAGGTTGACAGTG
	GPD1 terminator forward	KN1172	GAACAAAAACTCATCTCAGAAGAGGATCTGTAGAGGGGGTTACAGTAGCAGA
	GPD1 terminator reverse	KN0760	TCTCCAGCTCACATCCTCGGCACAAGGGTCTCAGGGATG
	NAT for CNAG_01664-Myc forward	KN0761	CATCCCTGAGACCCTTGTGCCGAGGATGTGAGCTGGAGA
	NAT for CNAG_01664-Myc reverse	KN1081	GGGGAAGTAAATTGTTCTAGGCTTTAACTAGCTTCCTGGTTTCAGAGACA
	CNAG_01664 right arm forward	KN1082	TGTCTCTGAAACCAGGAAGCTAGTTAAAGCCTAGAACAATTTACTTCCCC
	CNAG_01664 right arm reverse	KN1083	ATTTATCTTTTCCTCACATCGCC
CLN1-His$_6$	CNAG_06092 ORF forward	KN1047	CCGATTACGACCAGAGGTACTTTA
	CNAG_06092 ORF-His$_6$ reverse	KN1048	CTGCTACTGTAACCCCCTCTAGTGGTGATGGTGATGATGATTGCTTAGCCGTCTCATAAC
	GPD1 terminator forward	KN1049	GTTATGAGACGGCTAAGCAATCATCATCACCATCACCACTAGAGGGGGTTACAGTAGCAG
	GPD1 terminator reverse	KN0754	TCTCCAGCTCACATCCTCGGCACAAGGGTCTCAGGGATG
	NEO for CNAG_06092-His forward	KN0755	CATCCCTGAGACCCTTGTGCCGAGGATGTGAGCTGGAGA
	NEO for CNAG_06092-His reverse	KN0658	GGAATAACCTTAGCCATCGGGAAGAGATGTAGAAACTAGCTTCCTGG
	CNAG_06092 right arm forward	KN0659	CCAGGAAGCTAGTTTCTACATCTCTTCCCGATGGCTAAGGTTATTCC
	CNAG_06092 right arm reverse	KN0660	CGGGCGAAGAACGAAGAAGA
